# Gabapentin for the Management of Chronic Pelvic Pain in Women (GaPP1): A Pilot Randomised Controlled Trial

**DOI:** 10.1371/journal.pone.0153037

**Published:** 2016-04-12

**Authors:** Steff C. Lewis, Siladitya Bhattacharya, Olivia Wu, Katy Vincent, Stuart A. Jack, Hilary O. D. Critchley, Maureen A. Porter, Denise Cranley, John A. Wilson, Andrew W. Horne

**Affiliations:** 1 Centre for Population Health Sciences, University of Edinburgh, Edinburgh, Lothian, United Kingdom; 2 Applied Health Sciences, University of Aberdeen, Aberdeen, Grampian, United Kingdom; 3 Health Economics and Health Technology Assessment Institute of Health and Wellbeing, University of Glasgow, Glasgow, Lanarkshire, United Kingdom; 4 Nuffield Department of Obstetrics and Gynaecology, University of Oxford, John Radcliffe Hospital, Oxford, United Kingdom; 5 Gynaecology, Aberdeen Royal Infirmary, Aberdeen, Grampian, United Kingdom; 6 MRC Centre for Reproductive Health, University of Edinburgh, Edinburgh, Lothian, United Kingdom; 7 MRC Centre for Regenerative Medicine, University of Edinburgh, Edinburgh, Lothian, United Kingdom; 8 Department of Anaesthesia and Pain Medicine, Royal Infirmary of Edinburgh, Edinburgh, Lothian, United Kingdom; University of California Los Angeles, UNITED STATES

## Abstract

Chronic pelvic pain (CPP) affects 2.1–24% of women. Frequently, no underlying pathology is identified, and the pain is difficult to manage. Gabapentin is prescribed for CPP despite no robust evidence of efficacy. We performed a pilot trial in two UK centres to inform the planning of a future multicentre RCT to evaluate gabapentin in CPP management. Our primary objective was to determine levels of participant recruitment and retention. Secondary objectives included estimating potential effectiveness, acceptability to participants of trial methodology, and cost-effectiveness of gabapentin. Women with CPP and no obvious pelvic pathology were assigned to an increasing regimen of gabapentin (300-2700mg daily) or placebo. We calculated the proportion of eligible women randomised, and of randomised participants who were followed up to six months. The analyses by treatment group were by intention-to-treat. Interviews were conducted to evaluate women’s experiences of the trial. A probabilistic decision analytical model was used to estimate cost-effectiveness. Between September 2012–2013, 47 women (34% of those eligible) were randomised (22 to gabapentin, 25 to placebo), and 25 (53%) completed six-month follow-up. Participants on gabapentin had less pain (BPI difference 1.72 points, 95% CI:0.07–3.36), and an improvement in mood (HADS difference 4.35 points, 95% CI:1.97–6.73) at six months than those allocated placebo. The majority of participants described their trial experience favorably. At the UK threshold for willingness-to-pay, the probabilities of gabapentin or no treatment being cost-effective are similar. A pilot trial assessing gabapentin for CPP was feasible, but uncertainty remains, highlighting the need for a large definitive trial.

**Trial registration:**

Controlled-Trials.com ISRCTN45178534

## Introduction

The prevalence of chronic pelvic pain (CPP) ranges from 2.1% to 24% of the female population worldwide [1]. It is the reason for 20% of gynaecological consultations and causes a 45% reduction in work productivity [2]. The pathogenesis of the painful symptoms experienced by women with CPP is poorly understood. They can be associated with specific pathological processes, such as endometriosis, but up to 55% of women with CPP appear to have no obvious underlying pathology [3]. The management of CPP is difficult [4] because in the absence of underlying pathology, no established gynaecological treatments are available.

Gabapentin (a GABA analogue) is being increasingly prescribed in family medicine for CPP. With the support of the Scottish Primary Care Research Network, we surveyed a random group of Scottish family medicine practitioners to determine prescribing practice of gabapentin for CPP in women. Of the family medicine practitioners who responded to our survey, over 74% said that they would consider it as a treatment option for this condition. It is also recommended by some physicians for CPP in a multi-disciplinary setting, despite no evidence on which to base this recommendation, and no clear mechanism of action.

The efficacy of gabapentin has been documented for other chronic pain conditions: painful diabetic neuropathy, post-herpetic neuralgia, mixed neuropathic pain conditions, spinal cord injury and phantom limb pain [5]. In some of these trials, gabapentin has also been shown to improve sleep, mood and other elements of quality of life. To our knowledge, only one study has evaluated the use of gabapentin for CPP. This study (56 patients) compared gabapentin against amitriptyline for treatment of CPP and showed that gabapentin had greater efficacy (80% compared to 70% improvement in pain scores at 12 months) [6]. However, the significance of the effect on quality of life provided by gabapentin in the management of CPP was not evaluated and the study population included women with endometriosis and vulval conditions.

Ideally, a definitive evaluation of the efficacy of gabapentin in the management of CPP in women with no obvious underlying pathology requires a large carefully designed multicentre randomized controlled trial with appropriate inclusion and exclusion criteria. This was a pilot study to assess the processes that are vital to the success of such a trial.

## Materials and Methods

### Study protocol

The study protocol was published online prior to participant recruitment [7].

### Ethical approval

The study received ethical approval from the Scotland A Research Ethics Committee (REC 12/SS/0005) on 26 January 2012. All patients gave written informed consent.

### Participants

Women were recruited from gynaecology outpatient clinics, gynaecology wards and day surgery units between 10 September 2012 and 30 September 2013 (follow-up of last participant in completed 31 March 2014). They were eligible if they were aged between 18 and 50 years, had suffered from pelvic pain that was located within the true pelvis or between and below anterior iliac crests for greater than six months [8], had associated functional disability, had no obvious pelvic pathology at laparoscopy (between six months and two weeks prior to randomization) and were using effective contraception. They were excluded if they had known pelvic pathology such as endometriosis or an ovarian cyst, were already taking gabapentin or pregabalin, were due to undergo surgery in the next six months, had a history of significant renal impairment, were allergic to gabapentin, were breast feeding or were pregnant or planning pregnancy in the next six months.

### Sample size

We used a confidence interval approach to estimate the sample size required to estimate the proportion lost to follow up. We wished to show that the loss to follow-up was <20%. A 95% CI for 20% of 60 participants (12/60) is 11%–32%. We estimated that we would recruit approximately 3–4 participants per month from each centre and we aimed to recruit 60 participants over a nine-month recruitment period. However, due to staffing issues and consequent slow recruitment this period was extended to 12 months.

### Study design

This was a two-arm prospective parallel group 1:1 randomized controlled pilot trial, in two centres in Scotland, UK (NHS Lothian and NHS Grampian). Participants, all clinical staff, and those recording outcomes were blind to the allocated treatment until all outcome data had been recorded. After this point, individual participants were unblinded to their allocated treatment. The study team was not unblinded until after the database was finalized and locked.

### Interventions

After consent was obtained, eligible women were randomized by the clinical research team to either gabapentin or an identical-looking placebo using a web-based system that ensured allocation concealment. We used randomized blocks of size four, and stratified by centre. Participants were started on 300 mg gabapentin daily (or equivalent in placebo tablets) and increased in 300 mg increments each week until they reported a 50% pain reduction or side effects, up to a maximum dose of 2700 mg (or equivalent in placebo tablets). Participants were advised to continue with their allocated treatment for six months.

### Primary and secondary objectives

The primary objective was to determine whether it is possible to achieve acceptable levels of recruitment and retention in two UK centres. The secondary objectives were to estimate the effectiveness of gabapentin; the acceptability to participants of the proposed methods of recruitment, randomization, drug treatments and assessment tools; and to determine whether gabapentin is likely to be cost-effective given the current level of uncertainty and to ascertain whether it is appropriate to conduct further research to evaluate the potential value of gabapentin.

### Outcome measures

The clinical research team kept an electronic log of women who fulfilled the eligibility criteria, women who were invited to participate in the study, women recruited and women who left the trial early. Reasons for non-recruitment (e.g. non-eligibility, refusal to participate, administrative error), reasons for withdrawal and loss to follow-up were also recorded. A questionnaire was given to all participants at randomization (0 months) and at 3 months. This included a Visual Analogue Scale (VAS), Brief Pain Inventory (BPI), Pain Disability Questionnaire (PDQ), Hospital Anxiety and Depression Score (HADS), EQ5D Quality of Life (QoL), WHO QoL, Measure Yourself Medical Outcome Profile (MYMOP) and demographics. A further questionnaire was given to all participants at 6 months, which included the above and additional questions on whether they believed that they were receiving gabapentin or placebo and questions on acceptability of the allocated medication/treatment regimes (and compliance) and on the acceptability of the above data collection methods.

### Statistical analysis

A Statistical Analysis Plan was agreed prior to database lock. We calculated the proportion of eligible women randomized and of randomized participants who were followed up to six months, and provided estimates of these, with 95% confidence intervals. In addition, we determined the nature and number of unanswered questions in each questionnaire. The analyses by treatment group in this report are by intention-to-treat (ITT), in which all participants who were randomized into the study were analyzed in the group to which they were randomized, regardless of treatment received. A per protocol analysis was run in which participants who received less than two weeks of trial medication were removed. No participants received the opposite treatment to that allocated. The per protocol analysis removed three participants from the gabapentin arm, and eight from the placebo arm, but the results were very similar to those from the ITT analysis, and are therefore not presented here. For each outcome measure, the difference between treatment groups was calculated using analysis of covariance, adjusting for baseline scores. The EQ5D subscales were analyzed as changed versus not changed using Fisher’s Exact test, due to the small number of participants in some of the categories. Where there were missing data for an outcome variable, those records were removed from the analysis for that variable. Statistical analyses were performed using SAS software version 9.3 (SAS Institute, Cary NC, USA). There were no interim analyses. There was an eight-month extension to the funding, due to a late start date and slow recruitment. The study then continued until the planned date of finishing recruitment.

### Qualitative analysis

We had planned to invite women to participate in focus group discussions of the trial experience six months into the trial. However, this was not practically possible due to insufficient participant numbers (could not reach optimum size for a focus group i.e. six to eight participants). Instead, we offered individual semi-structured telephone interviews before unblinding that were recorded on a structured coding sheet. The interviews were analyzed thematically to identify the issues of importance to participants not covered in the questionnaires, their feelings about trial participation and experiences with prescribed medication.

### Health economic analysis

A probabilistic decision analytical model was used to estimate the potential cost-effectiveness of gabapentin compared with no active treatment, from the perspective of the NHS and personal social services (PSS). A decision tree model replicated the potential consequences of patients within the trial. Data relating to health outcomes (in terms of quality adjusted life years), healthcare resource use and direct medical costs were estimated from the results of the existing pilot study. The estimation of total costs took into account the costs of trial medication and GP contacts; quality adjusted life years associated were calculated based on the change from baseline method [9]. Gamma and beta distributions were applied to the cost and health utility parameters, respectively. Cost utility analysis was carried out and cost-effectiveness was expressed as incremental cost per quality-adjusted life years gained, where appropriate. An expected value of information analysis on the expected value of perfect information was also carried out to quantify potential value of further research based on the difference between expected net benefit with perfect information and existing information. For the model, it was assumed that the life of the technology (the period over which the decision of whether gabapentin compared with placebo, should be adopted remains relevant) is two years and the number of eligible participants has been estimated at 68,000 per annum in Scotland (based on a prevalence of 38/1000 women) [3].

## Results and Discussion

### Recruitment and retention

**Fig 1:**
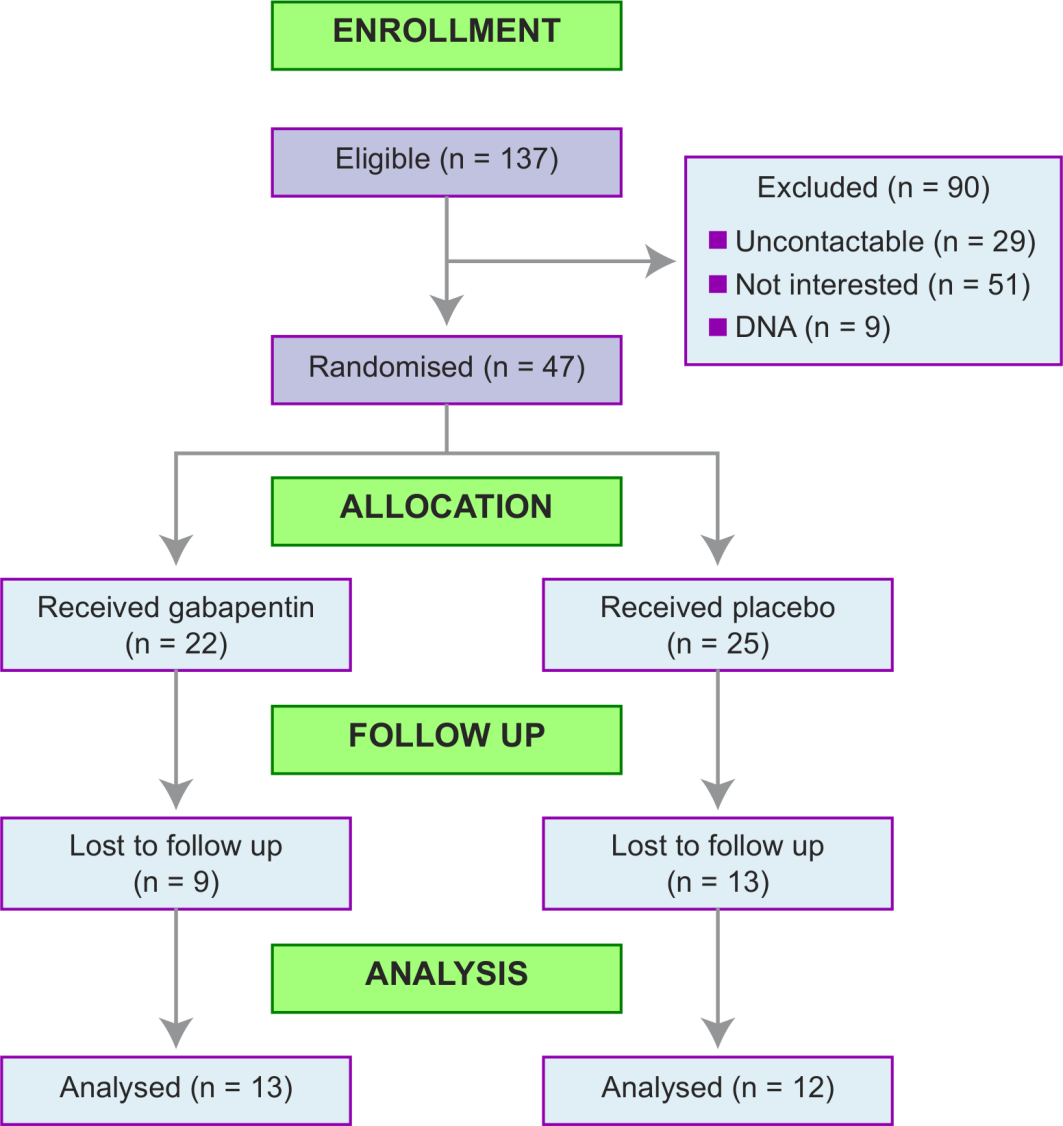
CONSORT flow chart for the GaPP1 trial. DNA = women who did not attend for screening.

From 137 eligible women, we randomized 47 (34%, 95% confidence interval 27%-43%) [Fig 1] between 10 September 2012 and 30 September 2013 (follow-up of last participant in completed 31 March 2014). Twenty-two women were allocated to gabapentin, and 25 to placebo. We were not able to capture all of the reasons why eligible women did not participate in the trial but examples of reasons recorded included ‘already tried gabapentin’, ‘not wanting to take daily pills’, and ‘symptoms improved since laparoscopy’. Baseline data were complete, and baseline characteristics were evenly distributed between the randomized groups [Table 1 and S1 Table].

**Table 1:** Characteristics at randomisation–categorical.

		Randomised treatment				All	
		Gabapentin		Placebo		Participants	
		N	%	N	%	N	%
Total number of participants randomised		22	100.0	25	100.0	47	100.0
Centre	Edinburgh	14	63.6	16	64.0	30	63.8
	Aberdeen	8	36.4	9	36.0	17	36.2
Deprivation category	1	1	4.5	1	4.0	2	4.3
	2	2	9.1	4	16.0	6	12.8
	3	5	22.7	6	24.0	11	23.4
	4	6	27.3	4	16.0	10	21.3
	5	5	22.7	4	16.0	9	19.1
	6	2	9.1	5	20.0	7	14.9
	7	0	0.0	1	4.0	1	2.1
	NA	1	4.5	0	0.0	1	2.1
Ethnicity	Caucasian	22	100.0	25	100.0	47	100.0
Parity	None	14	63.6	17	68.0	31	66.0
	1 or more	8	36.4	8	32.0	16	34.0
Contraception*	Any used	21	95.5	23	92.0	44	93.6
	Condoms	6	27.3	8	32.0	14	29.8
	COCP	2	9.1	4	16.0	6	12.8
	POP	1	4.5	1	4.0	2	4.3
	Depoprovera	1	4.5	3	12.0	4	8.5
	Nexplanon	2	9.1	0	0.0	2	4.3
	Mirena	7	31.8	8	32.0	15	31.9
	Copper IUD	0	0.0	0	0.0	0	0.0
	Sterilised	1	4.5	0	0.0	1	2.1
	Vasectomy	0	0.0	0	0.0	0	0.0
	Contraceptive patch	0	0.0	0	0.0	0	0.0
	Other	2	9.1	0	0.0	2	4.3
Marital Status	Single	12	54.5	17	68.0	29	61.7
	Separated	0	0.0	1	4.0	1	2.1
	Married	4	18.2	2	8.0	6	12.8
	Divorced	2	9.1	3	12.0	5	10.6
	Living as married	4	18.2	2	8.0	6	12.8
Highest education received	Secondary school	5	22.7	7	28.0	12	25.5
	College/University	17	77.3	18	72.0	35	74.5

*Women could use more than one type of contraception.

25/47 (53%) had data available from the final follow up [Fig 1]. 37/47 (79%) took at least two weeks of their medication [S2 Table]. The median number of weeks of medication taken was six (interquartile range 2 to 9). The number of participants with follow up data was equally distributed between the two treatment groups– 13 in the gabapentin group and 12 in the placebo group. One placebo participant who withdrew from treatment did have follow up data.

If women attempted to complete any follow up, then they generally did so fully, resulting in very few missing data points amongst the forms that were collected. Amongst those that answered at all, there were no missing data points for the VAS, PDQ, HADS and EQ5D. The BPI was fully completed by 42 (89%) women at baseline, 25 (96% of those who answered) at three months, and 21 (84% of those who answered) at six months. The WHO QOL was fully completed by 47 (100%) women at baseline, 26 (100% of those who answered) at three months, and 23 (92% of those who answered) at six months. In total, 20 (43%, 95% confidence interval 30%-57%) women fully completed all data collection forms and all of these took at least two weeks of their medication.

Six participants took their prescribed medication for less than two weeks: three changed their mind, one was withdrawn by the clinician due to a previous suicide attempt, and two withdrew due to side effects (one on gabapentin, one on placebo). Of those participants that took more than two weeks of treatment, ten withdrew. Three withdrew due to ‘lack of efficacy’ (one placebo, two gabapentin), two withdrew to try for a pregnancy, and five withdrew due to side effects (three gabapentin, two placebo). Four out of ten women given gabapentin, and seven out of ten women given placebo correctly guessed their treatment. This is similar to what would be expected by random chance alone (50%).

### Clinical efficacy

This was a feasibility study, and not large enough to produce reliable estimates of treatment effects. However, outcome measures were compared between groups, to give an indication of which measures might be likely to show a treatment effect in a larger study. Gabapentin was statistically significantly better than placebo using the BPI pain scale and HADS anxiety scale [Table 2]. Gabapentin was better than placebo, but with a wide confidence interval that could include clinical significant benefit or harm, for the VAS, BPI interference scale and HADS depression scale. Treatment effects were in opposite directions at three months and six months for the psychosocial scale of the PDQ—favoring placebo at three months and gabapentin at six months. However, these effects were not statistically significant. Gabapentin was worse than placebo, but with a wide confidence interval that could include clinical significant benefit or harm, for the function scale of the PDQ. There was no consistent pattern for the EQ-5D subscales, and no statistically significant results [S3 Table]. The MYMOP was not analyzed as the documentation of symptoms pre- and at the end of treatment was not accurately recorded by participants in the trial.

**Table 2:** Exploratory analysis of outcome data.

	Randomised treatment								
	Gabapentin			Placebo					
	N	mean	sd	N	mean	sd	Diff in means	95% CI	P
*VAS Q3—How strong was the pain during the past 4 weeks on average?*									
Baseline	13	6.6	1.8	13	6.3	1.9			
3m	13	4.2	2.7	13	5.1	2.3	1.13	-0.40 - +2.65	0.14
6m	13	3.6	2.4	12	4.5	2.3	0.98	-0.87 - +2.83	0.28
*BPI pain*									
Baseline	13	4.9	2.1	13	3.9	2.3			
3m	13	3.1	2.6	13	3.8	2.8	1.59	+0.03 - +3.14	0.05
6m	13	2.9	2.0	12	4.1	2.4	1.72	+0.07 - +3.36	0.04
*BPI interference*									
Baseline	13	4.1	2.9	13	3.3	2.9			
3m	13	3.1	2.5	13	3.2	2.7	0.57	-0.83 - +1.96	0.41
6m	13	3.2	2.9	12	3.1	1.9	0.00	-1.83 - +1.82	1.00
*Pain Disability Questionnaire psychosocial*									
Baseline	13	26.7	15.0	13	27.2	13.5			
3m	13	22.1	15.0	13	20.3	15.7	-2.27	-8.74 - +4.20	0.48
6m	13	16.2	14.8	12	20.2	16.1	2.34	-5.90 - +10.57	0.56
*Pain Disability Questionnaire function*									
Baseline	13	37.5	17.8	13	35.5	17.5			
3m	13	29.4	21.0	13	23.0	16.5	-5.25	-18.73 - +8.22	0.43
6m	13	23.9	25.3	12	20.3	14.8	-5.94	-20.93 - +9.05	0.42
*Hospital Anxiety and Depression Scale—anxiety*									
Baseline	13	10.2	5.5	13	8.1	3.9			
3m	13	8.1	5.4	13	8.2	4.2	1.98	-0.12 - +4.07	0.06
6m	13	7.5	5.7	12	9.8	5.3	4.35	+1.97 - +6.73	0.001
*Hospital Anxiety and Depression Scale—depression*									
Baseline	13	6.7	3.6	13	4.9	3.6			
3m	13	5.5	3.9	13	4.7	4.5	0.85	-1.41 - +3.10	0.45
6m	13	5.2	4.9	12	4.9	4.0	0.93	-1.81 - +3.66	0.49

The treatment effect is mean follow up (3m or 6m) value for placebo—gabapentin, adjusted for baseline values. Positive values indicate Gabapentin is better than placebo. Baseline data are shown for those with 3m data.

### Adverse events

17/22 (77%) gabapentin participants had an adverse event compared with 16/25 (64%) in the placebo group [Table 3]. The majority of these events were mild (15 in each group). There were two reported serious adverse events, both in the gabapentin group. However, both were exacerbations of CPP involving inpatient hospitalization that were not thought to be related to gabapentin.

**Table 3:** Adverse events. Number of participants with at least one adverse event, and severity of the worst event per participant.

		Randomised treatment				All	
		Gabapentin		Placebo		Participants	
		N	%	N	%	N	%
Total number of participants randomised		22	100.0	25	100.0	47	100.0
Had adverse event		17	77.3	16	64.0	33	70.2
Severity of worst event	Mild	15	68.2	15	60.0	30	63.8
	Moderate	0	0.0	1	4.0	1	2.1
	Serious	2	9.1	0	0.0	2	4.3

### Acceptability to participants of proposed methods of recruitment/randomisation/drug treatments/assessment tools

14 participants were interviewed for 10–15 minutes by an independent researcher before the six-month appointment at which they were unblinded. The majority of participants described their overall trial experience favorably, and rated it 1 or 2 on a 5-point scale from positive to negative. Positive responses were recorded for the recruitment approach used, paperwork burden for participants and the experience of randomization of participants. A suggestion of using electronic forms in a future trial was received favorably by participants. Negative feedback was largely related to trial medication, diary completion and communication issues. Some women experienced discomfort when swallowing large tablets; dose adjustment and managing side-effects were also problematic as participants did not always know what dose they were on and if symptoms were related to the drug or not. Some found the treatment diary too time consuming to complete successfully although most managed it. Communication issues were few but related to missed calls between participants and research nurses, and also a failure to read or understand fully the trial literature.

### Cost-effectiveness

**Fig 2:**
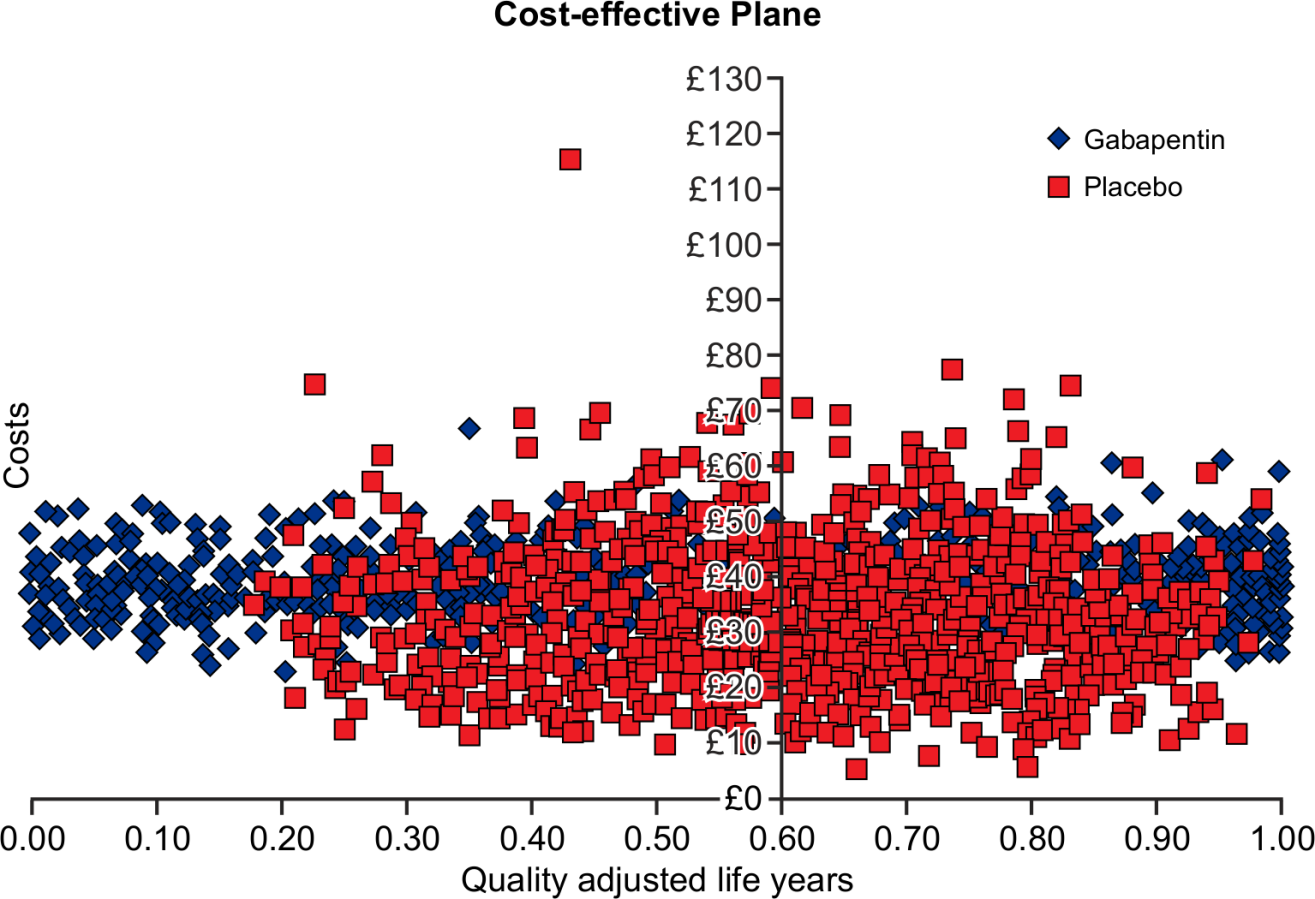
Cost-effectiveness plane.

**Fig 3:**
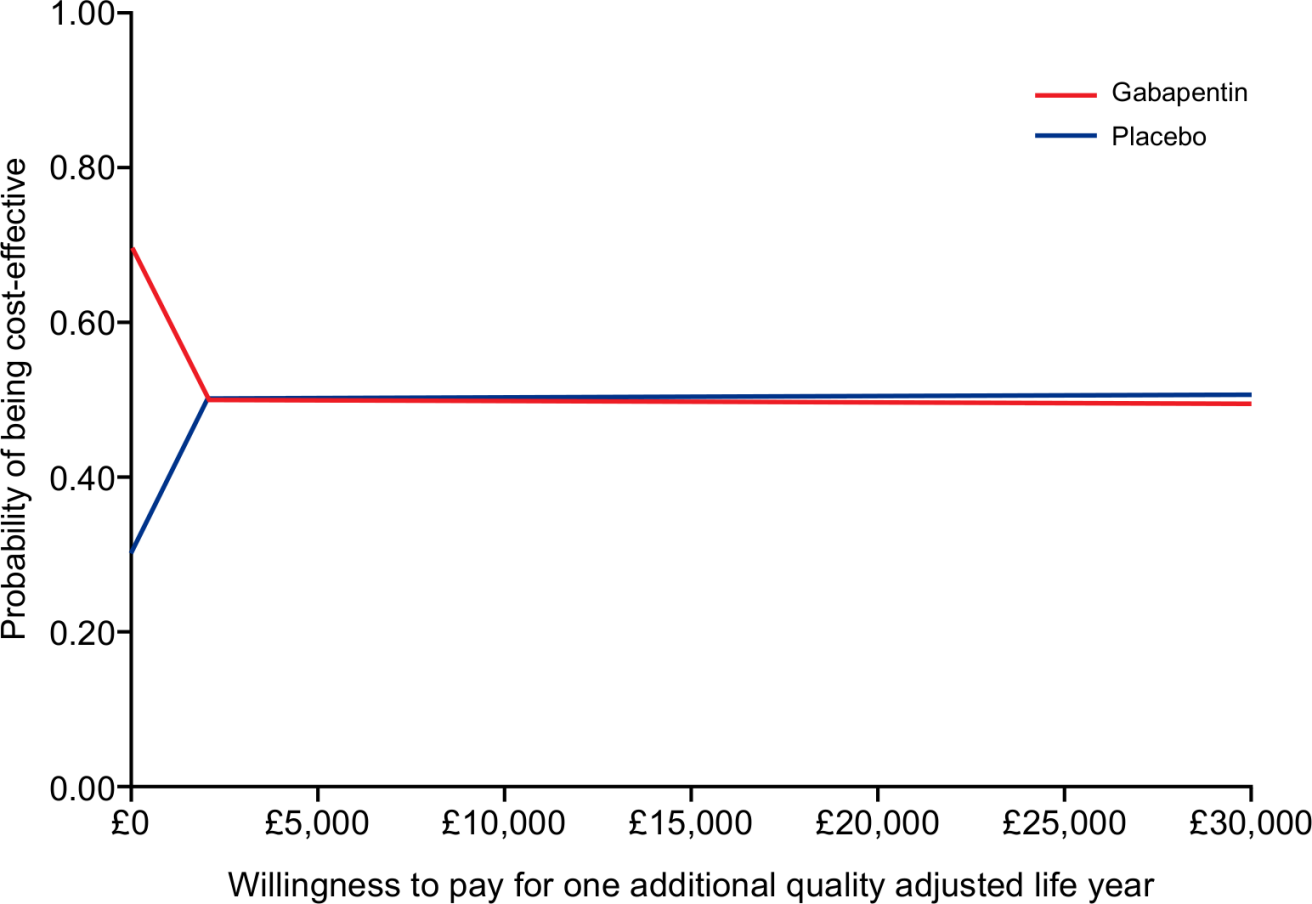
Cost-effectiveness acceptability curve.

The mean cost of gabapentin was £15.20 ($23) per participant, based on the assumption of weekly dose escalation as described in the “dose regimen” section earlier. It was assumed that no active treatment costs were incurred in the placebo arm. Costs associated with healthcare resource use, including GP visits and GP telephone consultations were greater in the placebo arm than the gabapentin arm [S4 Table]. As a result, the difference in mean total costs between the two arms was small (£19/$28.7 in the gabapentin arm compared with £17/$25.7 in the placebo arm). Gabapentin was also associated with lower QALYs compared with the placebo arm; however, the difference in QALYs is small [S4 Table]. Gabapentin is dominated by placebo. Probabilistic sensitivity analysis based on 1000 simulations gave similar results, but showed much uncertainty related to the parameter estimates [Fig 2]. The cost effectiveness acceptability curve showed that at willingness-to-pay of £20,000 ($30,235) to £30,000 ($45,350), gabapentin has a similar probability of being cost-effective than placebo at a probability of 51% [Fig 3]. The expected value of perfect information analysis indicated that future research is potentially cost-effective. At a willingness-to-pay threshold of £20,000, additional research is potentially cost effective if research is not proposed to exceed a cost of £325 million ($463 million).

## Discussion

Our pilot trial supports the feasibility of a future large multicentre randomised controlled trial to determine the efficacy of gabapentin in the management of CPP in women and provides preliminary evidence that gabapentin could be effective.

Feasibility is supported by the fact that, over a one year recruitment period, we were able to randomize 47 women (34% of those eligible) to gabapentin or matched placebo and obtain completed six month follow up in 25 women (53%). Regarding effectiveness, analysis of one of the pain scores (BPI questionnaire) used in the pilot indicates that participants randomized to gabapentin perform better than placebo: 1.72 points, 95% CI: 0.07–3.36 at six months (p = 0.04). In addition, analysis of the HADS anxiety scale used in the pilot supports an improvement in mood: 4.35 points, 95% CI: 1.97–6.73 at six months (p = 0.001).

Analysis of the interview data regarding the delivery of the trial was generally favorable. Most women were happy with the content, tone, and length of the written information received, and the provision of verbal information was a valued addition. Negative feedback was largely related to trial medication (large size of tablets), diary completion and communication issues. Whilst the delivery of the medication cannot be altered, the other feedback has alerted us to the need to develop the option of an online treatment diary and encourage better communication amongst the research staff (email, text) in the future trial.

Cost-effectiveness analysis showed that gabapentin and placebo has similar probabilities of being cost-effective at they typical threshold for willingness to pay in the UK. However, this is associated with high level of uncertainty; consequently, the value of future research to better quantify the associated costs and clinical benefits is high.

The level of recruitment in our pilot (34% of eligible women) was less than we had predicted (50%). However, these data have allowed us to estimate recruitment more accurately in our proposed future trial, and determine the number of centres (eight) and the recruitment period that we will require (24 months). We have used information on efficacy to inform the power calculation for the future trial (300 women).

A lower than expected proportion of patients followed up to six months (53%) has also alerted us to the need to improve retention in our future trial. Thus, we will ensure that all future trial centre research nurses understand that when a participant stops medication they should be encouraged to provide follow-up data. We will monitor this very closely, and keep in close contact with all trial centre staff. We will contact participants by other appropriate methods (email, telephone) to encourage replies. We plan to pioneer a database text message reminder system for dose titration in the trial and for reporting of pain scores. We will engage a commercial provider who can deliver reminder texts, e.g. for increasing the dose, and collect responses to text messages requesting pain scores. Integration with the trial database will enable automated texts to be sent according to a schedule determined by the consent and randomization dates. We will provide an online facility for any participants wishing to complete questionnaire booklets online. In addition, we will further analyze the current study to determine which factors predict ‘missingness’, so that we can plan appropriate missing data methodology in the statistical analysis of the full study.

We are now ready to capitalize on our pilot study and have secured funding to perform a definitive large multicentre double-blind randomised controlled trial to determine the true efficacy of gabapentin in the management of women with CPP (EudraCT 2014-005035-13; for more information go to the trial website http://www.birmingham.ac.uk/research/activity/mds/trials/bctu/trials/womens/GaPP2/index.aspx).

## Supporting Information

S1 Table (DOCX)

S2 Table (DOCX)

S3 Table (DOCX)

S4 Table (DOCX)

S1 Text Published trial protocol paper. (PDF)

S2 Text Consort checklist. (DOC)

S3 Text Full Trial protocol (approved by ethics committee). (PDF)
