# Supplementary material for: Gabapentin for the Management of Chronic Pelvic Pain in Women (GaPP1): A Pilot Randomised Controlled Trial
Source: PLoS One. 2016 Apr 12;11(4):e0153037. doi: 10.1371/journal.pone.0153037 (PMC4829183; doi:10.1371/journal.pone.0153037)
Supplement: S1 Table — (DOCX) [file pone.0153037.s001.docx]

**S1 Table.** Characteristics at randomisation – continuous.

|  | Randomised Treatment | N | Mean | SD | Maximum | Upper quartile | Median | Lower quartile | Minimum |
| --- | --- | --- | --- | --- | --- | --- | --- | --- | --- |
| Age | Gabapentin | 22 | 26.8 | 7.7 | 43 | 33 | 26 | 20 | 18 |
| (years) | Placebo | 25 | 27.1 | 5.8 | 42 | 30 | 27 | 24 | 18 |
|  | All Participants | 47 | 26.9 | 6.7 | 43 | 30 | 26 | 22 | 18 |
| BMI | Gabapentin | 22 | 25.9 | 4.1 | 34.0 | 29.0 | 24.8 | 23.0 | 19.5 |
| (kg/m2) | Placebo | 25 | 25.9 | 4.4 | 34.1 | 29.1 | 25.7 | 22.5 | 18.4 |
|  | All Participants | 47 | 25.9 | 4.2 | 34.1 | 29.1 | 25.6 | 22.8 | 18.4 |
