# Supplementary material for: Gabapentin for the Management of Chronic Pelvic Pain in Women (GaPP1): A Pilot Randomised Controlled Trial
Source: PLoS One. 2016 Apr 12;11(4):e0153037. doi: 10.1371/journal.pone.0153037 (PMC4829183; doi:10.1371/journal.pone.0153037)
Supplement: S2 Table — (DOCX) [file pone.0153037.s002.docx]

**S2 Table .** Compliance with treatment.

|  |  | **Randomised treatment** | | | | **All** | |
| --- | --- | --- | --- | --- | --- | --- | --- |
|  |  | **Gabapentin** | | **Placebo** | | **Participants** | |
|  |  | **N** | **%** | **N** | **%** | **N** | **%** |
| Total number of participants randomised | | 22 | 100.0 | 25 | 100.0 | 47 | 100.0 |
| Took 1st 2 weeks of medication |  | 19 | 86.4 | 17 | 68.0 | 36 | 76.6 |
| Took any 2 weeks of medication |  | 19 | 86.4 | 18 | 72.0 | 37 | 78.7 |
| Fully completed all 6m forms |  | 11 | 50.0 | 9 | 36.0 | 20 | 42.6 |
| Fully completed all 6m forms and took 1st 2 weeks of medication |  | 11 | 50.0 | 9 | 36.0 | 20 | 42.6 |

** NB. Fig 1 includes participants who were in ‘ANY’ 6m analysis, and Table 3 includes participants who completed ALL 6m forms with no missing data.*
