# Supplementary material for: Gabapentin for the Management of Chronic Pelvic Pain in Women (GaPP1): A Pilot Randomised Controlled Trial
Source: PLoS One. 2016 Apr 12;11(4):e0153037. doi: 10.1371/journal.pone.0153037 (PMC4829183; doi:10.1371/journal.pone.0153037)
Supplement: S3 Table — (DOCX) [file pone.0153037.s003.docx]

**S3 Table .** Exploratory analysis of EQ 5D.

|  | **Randomised treatment** | | | |  |
| --- | --- | --- | --- | --- | --- |
|  | **Gabapentin** | | **Placebo** | |  |
|  | **N** | **%** | **N** | **%** | **P** |
| *EQ5D - mobility* |  |  |  |  |  |
| Improved at 3m | 6 | 46% | 4 | 31% | 0.69 |
| Improved at 6m | 7 | 54% | 3 | 25% | 0.23 |
| *EQ5D – self care* |  |  |  |  |  |
| Improved at 3m | 1 | 8% | 2 | 15% | 1.00 |
| Improved at 6m | 0 | 0% | 3 | 25% | 0.10 |
| *EQ5D - activities* |  |  |  |  |  |
| Improved at 3m | 3 | 23% | 7 | 54% | 0.23 |
| Improved at 6m | 6 | 46% | 4 | 33% | 0.69 |
| *EQ5D - pain* |  |  |  |  |  |
| Improved at 3m | 5 | 38% | 7 | 54% | 0.70 |
| Improved at 6m | 5 | 38% | 4 | 33% | 1.00 |
| *EQ5D - anxiety* |  |  |  |  |  |
| Improved at 3m | 6 | 50% | 4 | 31% | 0.69 |
| Improved at 6m | 6 | 46% | 3 | 25% | 0.41 |
