# Supplementary material for: Gabapentin for the Management of Chronic Pelvic Pain in Women (GaPP1): A Pilot Randomised Controlled Trial
Source: PLoS One. 2016 Apr 12;11(4):e0153037. doi: 10.1371/journal.pone.0153037 (PMC4829183; doi:10.1371/journal.pone.0153037)
Supplement: S4 Table — (DOCX) [file pone.0153037.s004.docx]

**S4 Table .** Resource use, costs and health utilities.

|  | **Gabapentin** |  | **Placebo** |  |
| --- | --- | --- | --- | --- |
| **Resource Use** | **N** | **No. of Contacts** | **N** | **No. of Contacts** |
| GP visits | 2 | 2 | 4 | 8 |
| GP telephone consultations | 0 | - | 1 | 1 |
|  |  |  |  |  |
| **Costs** |  | **Mean±SD** |  | **Mean±SD** |
| Trial medication | 22 | £15.23±14.39 | 25 | - |
| GP visits (£45 per consultation)* |  | £4.10±13.24 |  | £14.40±40.50 |
| GP call (£27 per consultation)* |  | - |  | £2.16±10.80 |
|  |  |  |  |  |
| **Basecase Results** |  |  |  |  |
| Costs |  | £19.32±19.93 |  | £16.56±43.53 |
| QALYs |  | 0.57±0.30 |  | 0.61±0.17 |
|  |  |  |  |  |

* Unit costs are taken from Unit costs of health and social care 2013, Personal Social Services Research Unit, Kent.
