# Supplementary material for: Gabapentin for the Management of Chronic Pelvic Pain in Women (GaPP1): A Pilot Randomised Controlled Trial
Source: PLoS One. 2016 Apr 12;11(4):e0153037. doi: 10.1371/journal.pone.0153037 (PMC4829183; doi:10.1371/journal.pone.0153037)
Supplement: S3 Text — (PDF) [file pone.0153037.s007.pdf]

GaPP PROTOCOL

GaPP: A pilot randomised controlled trial of the efficacy and mechanism of action of gabapentin for the management of chronic pelvic pain in women

Investigators

Dr Andrew Horne, University of Edinburgh

Prof Siladitya Bhattacharya, University of Aberdeen

Co-sponsors

University of Edinburgh and NHS Lothian
ACCORD
The Queen's Medical Research Institute
47 Little France Crescent
Edinburgh EH16 4TJ

Funder

Chief Scientist Office (Scotland)

Funding Reference Number

CZH/4/688

Chief Investigator

Dr Andrew Horne

EudraCT Number

2011-005494-22

REC Number

12/SS/0005

ISRCTN Number

ISCTRN70960777

Version Number and Date

Version 5_11th December 2012

Coordinating Centre

MRC Centre for Reproductive Health
The University of Edinburgh
The Queen's Medical Research Institute
47 Little France Crescent
Edinburgh EH16 4TJ

Co-sponsor Representative

Dr Lynn Morrice
University of Edinburgh and NHS Lothian
ACCORD
The Queen's Medical Research Institute
47 Little France Crescent
Edinburgh EH16 4TJ

Tel: 0131 242 9461
Fax: 0131 242 9447
Email: lynn.morrice@ed.ac.uk

Trial Manager
Mrs Ann Doust
Room S7128
2nd Floor Simpson Centre for Reproductive Biology
Royal Infirmary of Edinburgh
51 Little France Crescent
Edinburgh EH16 4SA
Tel: 0131 242 9492
Fax: 0131 242 2686
Email: ann.doust@ed.ac.uk

Trial Statistician
Dr Steff Lewis
Centre for Population Health Sciences
University of Edinburgh Medical School
Teviot Place
Edinburgh EH8 9AG
Tel: 0131 650 3198
Fax:
Email: steff.lewis@ed.ac.uk

Co-Investigator
Prof Siladitya Bhattacharya
Division of Applied Health Sciences
School of Medicine and Dentistry
University of Aberdeen
Aberdeen Maternity Hospital
Foresterhill
Aberdeen AB25 2ZD
Tel: +44 1224 550590
Fax: +44 1224 559948
Email: s.bhattacharya@abdn.ac.uk

Co-Investigator
Prof Hilary Critchley
MRC Centre for Reproductive Health
The University of Edinburgh
The Queen's Medical Research Institute
47 Little France Crescent
Edinburgh EH16 4TJ
Tel: 0131 242 6439
Fax: 0131 242 6441
Email: hilary.critchley@ed.ac.uk

Co-Investigator
Dr Cyril Pernet
SFC Brain Imaging Research Centre
Western General Hospital
Crewe Road
Edinburgh
Tel: 0131 537 3661
Fax:
Email: cyril.pernet@ed.ac.uk

Co-Investigator
Dr John Wilson
Dept of Anaesthesia, Critical Care and Pain Medicine
Royal Infirmary of Edinburgh
Little France Crescent
Edinburgh EH16 4SA
Tel: 0131 242 3192
Fax:
Email: john.wilson@luht.scot.nhs.uk

Co-Investigator
Dr Olivia Wu
Centre for Population and Health Sciences
University of Glasgow
1 Lilybank Gardens
Glasgow G12 8RZ
Tel: 0141 330 3296
Fax: 0141 330 5018
Email: Olivia.wu@glasgow.ac.uk

Co-Investigator
Dr Maureen Porter
Division of Applied Health Sciences
University of Aberdeen
Aberdeen Maternity Hospital
Aberdeen
Tel: 01224 554875
Fax:
Email: m.a.porter@abdn.ac.uk

Co-Investigator
Dr Stuart Jack
Department of Gynaecology
Aberdeen Royal Infirmary
Aberdeen AB25 2ZN
Tel: 01224 553471
Fax:
Email: sjackhome@btinternet.com

Participating Sites

Principal Investigator NHS Lothian
Dr Andrew Horne
MRC Centre for Reproductive Health
The University of Edinburgh
The Queen's Medical Research Institute
47 Little France Crescent
Edinburgh EH16 4TJ
Tel: 0131 242 6609
Fax: 0131 242 6441
Email: andrew.horne@ed.ac.uk

Principal Investigator NHS Grampian
Prof Siladitya Bhattacharya
School of Medicine and Dentistry
University of Aberdeen
Aberdeen Maternity Hospital
Foresterhill
Aberdeen AB25 2ZD
Tel: +44 1224 550590
Fax: +44 1224 559948
Email: s.bhattacharya@abdn.ac.uk

Research Sister
Mrs Helen Dewart
Room G7138
Simpson Centre for Reproductive Health
Royal Infirmary of Edinburgh
51 Little France Crescent
Edinburgh EH16 4SA
Tel: 0131 242 2678
Fax: 0131 242 2686
Email: helen.dewart@ed.ac.uk

Research Nurse
Roslyn Campbell, Research Nurse
GUM Clinic
Woolmanhill Hospital
Aberdeen AB25 1LD
Tel: 01224 555405
e-mail: r.mchardy@nhs.net

Index

Contents 5-6
Protocol approval 7
List of abbreviations 8
Summary 9

1 INTRODUCTION 10
- 1.1 BACKGROUND 10
- 1.2 RATIONALE FOR STUDY 10

2 STUDY OBJECTIVES 10
- 2.1 OBJECTIVES 10
  - 2.1.1 Primary Objective 10
  - 2.1.2 Secondary Objectives 10
- 2.2 ENDPOINTS 10
  - 2.2.1 Primary Endpoint 10
  - 2.2.2 Secondary Endpoints 10

3 STUDY DESIGN 10
- 4.1 NUMBER OF PARTICIPANTS 11
- 4.2 INCLUSION CRITERIA 11
- 4.3 EXCLUSION CRITERIA 11

5 PARTICIPANT SELECTION AND ENROLMENT 11
- 5.2 CONSENTING PARTICIPANTS 11
- 5.3 SCREENING FOR ELIGIBILITY 11
- 5.4 INELIGIBLE AND NON-RECRUITED PARTICIPANTS 11
- 5.5 RANDOMISATION 11
  - 5.5.1 Randomisation Procedures 11
  - 5.5.2 Treatment Allocation 11
  - 5.5.3 Emergency Unblinding Procedures 11
  - 5.5.4 Withdrawal of Study Participants 12

6 INVESTIGATIONAL MEDICINAL PRODUCT AND PLACEBO 12
- 6.1 STUDY DRUG 12
  - 6.1.1 Study Drug Identification 12
  - 6.1.2 Study Drug Manufacturer 12
  - 6.1.3 Marketing Authorisation Holder 12
  - 6.1.4 Labelling and Packaging 12
  - 6.1.5 Storage 12
  - 6.1.6 Summary of Product Characteristics or Investigators Brochure 12
- 6.2 PLACEBO 12
- 6.3 DOSING REGIME 12
- 6.4 DOSE CHANGES 12
- 6.5 PARTICIPANT COMPLIANCE 12
- 6.6 OVERDOSE 13
- 6.7 OTHER MEDICATIONS 13
  - 6.7.1 Non-Investigational Medicinal Products 13
  - 6.7.2 Permitted Medications 13
  - 6.7.3 Prohibited Medications 14

7 STUDY ASSESSMENTS 15
- 7.1 SAFETY ASSESSMENTS 15
- 7.1 SAFETY ASSESSMENTS 15
- 7.2 STUDY ASSESSMENTS 15
- 7.2 STUDY ASSESSMENTS 15

8 DATA COLLECTION 16

9 STATISTICS AND DATA ANALYSIS **17**
9.1 SAMPLE SIZE CALCULATION 17
9.2 PROPOSED ANALYSES 17
10 ADVERSE EVENTS **18**
10.1 DEFINITIONS 18
10.2 DETECTING AEs AND SAEs 18
10.3 RECORDING AEs AND SAEs 18
10.4 ASSESSMENT OF AEs AND SAEs 19
10.4.1 Assessment of Seriousness 19
10.4.2 Assessment of Causality 19
10.4.3 Assessment of Severity 19
10.4.4 Assessment of Expectedness 19
10.5 REPORTING OF SAEs/SARs/SUSARs 19
10.6 REGULATORY REPORTING REQUIREMENTS 19
10.7 FOLLOW UP PROCEDURES 20
11. PREGNANCY **20**
12 TRIAL MANAGEMENT AND OVERSIGHT ARRANGEMENTS **20**
12.1 TRIAL MANAGEMENT GROUP 20
12.2 TRIAL STEERING COMMITTEE 20
12.3 DATA MONITORING COMMITTEE 20
12.4 INSPECTION OF RECORDS 20
12.5 RISK ASSESSMENT 20
12.6 STUDY MONITORING AND AUDIT 20
13 GOOD CLINICAL PRACTICE **21**
13.1 ETHICAL CONDUCT 21
13.2 REGULATORY COMPLIANCE 21
13.3 INVESTIGATOR RESPONSIBILITIES 21
13.3.1 Informed Consent 21
13.3.2 Study Site Staff 21
13.3.3 Data Recording 21
13.3.4 Investigator Documentation 21
13.3.5 GCP Training 21
13.3.6 Confidentiality 21
13.3.7 Data Protection 21
14 STUDY CONDUCT RESPONSIBILITIES **22**
14.1 PROTOCOL AMENDMENTS 22
14.2 PROTOCOL VIOLATIONS AND DEVIATIONS 22
14.3 SERIOUS BREACH REQUIREMENTS 22
14.4 STUDY RECORD RETENTION 22
14.5 END OF STUDY 22
14.6 CONTINUATION OF DRUG FOLLOWING THE END OF STUDY 22
14.7 INSURANCE AND INDEMNITY 22
15 REPORTING, PUBLICATIONS AND NOTIFICATION OF RESULTS **23**
15.1 AUTHORSHIP POLICY 23
15.2 PUBLICATION 23
15.3 PEER REVIEW 23
16 REFERENCES **24**
APPENDIX 1: Summary of Product Characteristics 25
APPENDIX 2: Trial Steering Committee 26
APPENDIX 3: Data Monitoring Committee 26
APPENDIX 4: World Medical Association Declaration Of Helsinki 27
APPENDIX 5: Study Flowchart 28
APPENDIX 6: Flowchart 29

Protocol Approval

GaPP: A pilot randomised controlled trial of the efficacy and mechanism of action of gabapentin for the management of chronic pelvic pain in women

EudraCT number 2011-005494-22

Signatures

Chief Investigator	Signature ______________________	Date ____________
Trial Statistician	Signature ______________________	Date ____________
Sponsor's Representative	Signature ______________________	Date ____________
Principal Investigator	Signature ______________________	Date ____________

List of Abbreviations

GCP	Good Clinical Practice
ICH	International Conference on Harmonisation
IMP	Investigational Medicinal Product
ISF	Investigator Site File
TMF	Trial Master File
SOP	Standard Operating Procedure
CRF	Case Report Form
AE	Adverse Event
SAE	Serious Adverse Event
AR	Adverse Reaction
SAR	Serious Adverse Reaction
UAR	Unexpected Adverse Reaction
SUSAR	Suspected Unexpected Serious Adverse Reaction
ACCORD	Academic and Clinical Central Office for Research & Development - Joint office for University of Edinburgh and NHS Lothian

Summary

Chronic pelvic pain (CPP) affects >1 million UK women. Annual healthcare costs are estimated at >£150 million. Proven interventions for CPP are limited and treatment is often unsatisfactory. Gabapentin is increasingly prescribed due to reports of effectiveness in other chronic pain conditions, but there are insufficient data supporting value in CPP specifically. The mechanism of action of gabapentin in CPP is also unclear. Given the prevalence and costs of CPP, we believe a large multi-centre placebo-controlled double-blind randomised-controlled trial (RCT) to evaluate the efficacy of gabapentin in management of CPP is required along with exploration of its mechanism of action. The focus of this study is a pilot to inform planning of a future RCT. We aim to recruit 60 women with CPP in NHS Lothian and NHS Grampian and randomise them to gabapentin or placebo. Response to treatment will be monitored by questionnaire at 3 and 6 months. A random subset of 24 Edinburgh women will undergo functional magnetic resonance imaging of the brain at 3 months. Our primary objective is to assess recruitment and retention rates and to obtain data to refine the research methodology for the RCT. We also aim to perform a pre-trial cost-effectiveness assessment of treatment with gabapentin.

1 INTRODUCTION

1.1 BACKGROUND

Chronic pelvic pain (CPP) affects over 1 million women in the UK (Latthe 2006); Daniels & Khan 2010). It is the reason for 20% of gynaecological consultations and causes a 45% reduction in work productivity (Cheong 2006). The annual cost for caring for UK women with CPP has been estimated at £154 million (Cheong 2006). The pathogenesis of the painful symptoms experienced by women with CPP is poorly understood. They are associated with specific pathological processes, such as endometriosis, but up to 55% of women with CPP appear to have no obvious underlying pathology (Daniels & Khan 2010). The management of CPP is difficult (Stones & Mountfield 2000) because in the absence of underlying pathology, no established gynaecological treatments are available.

1.2 RATIONALE FOR STUDY

Gabapentin (a GABA analogue) is being increasingly prescribed in general practice for CPP. It is also recommended by some practitioners as a treatment of choice for CPP in a multi-disciplinary setting, despite no evidence on which to base this recommendation, and no clear mechanism of action. To our knowledge, only one study has evaluated the use of gabapentin for CPP. This small study (56 patients) compared gabapentin against amitriptyline for treatment of CPP and showed that gabapentin had greater efficacy (80% compared to 70% improvement in pain scores at 12 months) (Sator-Katzenschlager 2005). Unfortunately, this study had no placebo arm and the significance of the effect on quality of life provided by gabapentin in the management of CPP was not evaluated. Nevertheless, the efficacy of gabapentin has been documented for other chronic pain conditions: painful diabetic neuropathy, postherpetic neuralgia, mixed neuropathic pain conditions, spinal-cord injury and phantom limb pain (Wiffen 2005). The number needed to treat for improvement in all trials with evaluable data is 4.3 (95% CI 3.5 to 5.7). In some of these trials, gabapentin also improved sleep, mood and other elements of quality of life.

Current understanding of the mechanism of action of gabapentin has relied on studies in non-specific disease models and animals using functional magnetic resonance imaging (fMRI) of the brain. This modality has been successfully employed to investigate nocioceptive processing in humans and has shown that gabapentin is effective in modulating nocioceptive transmission when central sensitisation is present (Iannetti 2005). In anaesthetised rats, fMRI has shown that gabapentin produces significant and discrete changes in brain activation (Governo 2008). However, a recent review has highlighted the need for studies of nocioceptive processing, using fMRI, to further understanding of CPP (Vincent 2009). We believe that fMRI could also be used to investigate the mechanism of action of gabapentin in CPP, leading to further understanding of the pathophysiology of painful symptoms of CPP and the identification of novel treatment strategies.

Ideally, a definitive evaluation of the efficacy and mechanism of action of gabapentin in the management of CPP with no obvious underlying pathology requires a large multicentre randomised controlled trial (RCT). This is a pilot study to assess the processes that are vital to the success of such a trial.

2 STUDY OBJECTIVES

2.1 OBJECTIVES

2.1.1 Primary Objective

The primary objective is to determine whether it is possible to achieve acceptable recruitment and retention rates in two centres (NHS Lothian and NHS Grampian) within defined inclusion/exclusion criteria

2.1.2 Secondary Objectives

1. To determine the effectiveness and acceptability to patients of the proposed methods of recruitment, randomisation, drug treatments, use of brain fMRI and follow-up
2. To determine whether fMRI of the brain a sensitive approach to determine the mechanism of action of gabapentin in the management of CPP
3. To determine whether gabapentin is likely to be cost effective given the current level of evidence and uncertainty, and to ascertain what further evidence is needed for the evaluation of gabapentin

2.2 ENDPOINTS

2.2.1 Primary Endpoints

1. The proportion of eligible patients randomised into the study.
2. The proportion of randomised patients who take all their medication and fully complete questionnaires at final follow up.

2.2.2 Secondary Endpoints

The remaining data (effectiveness and acceptability of proposed methods of recruitment, randomisation, drug treatments and questionnaires use of brain fMRI and follow-up; and sensitivity of brain fMRI to study the effect of gabapentin in the management of CPP) will be used to refine the research methodology for the RCT.

3 STUDY DESIGN

A two-arm parallel randomised controlled pilot trial. This will be a two centre pilot study with sites in NHS Lothian and NHS Grampian. We will recruit 60 patients over approximately nine months. After randomisation the participants will receive treatment for six months. Participants and the health care team will be unblinded at the end of their treatment.

4 STUDY

4.1 NUMBER OF PARTICIPANTS

60 women aged 18-50 with a history of pelvic pain (cyclical or non-cyclical) and/or dyspareunia will be invited to participate in the trial.

4.2 INCLUSION CRITERIA

- Women aged between 18-50
- Pelvic pain of > 6 months
- Pain located within the true pelvis or between and below anterior iliac crests, associated functional disability
- No obvious pelvic pathology at laparoscopy (<3 years and >2 weeks ago)
- Using effective contraception

4.3 EXCLUSION CRITERIA

- Known pelvic pathology eg endometriosis, cyst
- Taking gabapentin or pregabalin or any restricted medications
- Due to undergo surgery in the next 6 months
- History of renal impairment
- Allergic to gabapentin
- Breast feeding
- Pregnancy or planning pregnancy in the next 6 months

5 PARTICIPANT SELECTION AND ENROLMENT
(See flowchart for recruitment strategy – Appendix 5)

5.1 IDENTIFYING PARTICIPANTS

Women will be identified by their clinician and asked if they will talk to a member of the clinical research team. If they agree, they will then be given a patient information sheet and will be asked if they are happy to be contacted to get their decision on whether they wish to participate. This will give them ample time to read the patient information sheet, ask any questions and make an informed choice of whether to participate or not.

5.2 CONSENTING PARTICIPANTS

Eligible participants will be consented by a member of the clinical research team.

5.3 SCREENING FOR ELIGIBILITY

If participants express an interest in the study they will be assessed to determine whether they fulfil all the potential eligibility criteria.

5.4 INELIGIBLE AND NON-RECRUITED PARTICIPANTS

The following information will be monitored and collected:

- Demographic information (age, current medication)
- Reason for not entering the randomisation phase

5.5 RANDOMISATION

5.5.1 Randomisation Procedures

Eligible women will be randomised to either gabapentin or placebo using a web-based system. Women will be stratified by centre (Edinburgh and Aberdeen). We will use randomised blocks of varying sizes.

5.5.2 Treatment Allocation

After randomisation, participants will be allocated an oral treatment pack containing either gabapentin or placebo, both of identical appearance. There will be a drug protocol to follow in their treatment diaries. The same protocol will be used for the placebo. The participants will be allowed to use other medication (including analgesics) throughout the study period. The participant will be advised to use effective contraception throughout the study and for one week after.

5.5.3 Emergency Unblinding Procedures

The blinding code will only be broken in emergency situations for reasons of patient safety, where knowledge of the treatment administered is necessary for the treatment of a serious adverse event or when required by local regulatory authorities. Participants whose randomisation codes are broken will cease treatment with the study drug but will continue to be followed up. The Investigator must document the reason for breaking the blind in the participant's source documents and in the Investigator Site Master File.

5.5.4 Withdrawal of Study Participants

Participants may discontinue from the trial at any time at their own request, or they may be withdrawn at any time at the discretion of the Investigator or sponsor for safety, behavioural or administrative reasons. Withdrawal may be from treatment or from the whole trial. With permission all data obtained up to point of withdrawal will be used. If a participant does not return for a scheduled visit, every effort will be made to contact the participant. In any circumstance, every effort will be made to document subject outcome, if possible. The investigator will inquire about the reason for withdrawal, request that the subject return all unused investigational products, return for a final visit, if applicable, and follow-up with the subject regarding any unresolved adverse events.

If the participant withdraws from treatment then she will be unblinded and if on gabapentin she will be advised to reduce the dose according to a recognised dose reduction protocol.

If the participant discontinues from the trial and also withdraws consent for the disclosure of future information, no further evaluations will be performed and no additional data will be collected. The sponsor may retain and continue to use any data collected before such withdrawal of consent. If the participant shows signs of acute pancreatitis or falls pregnant, they will be withdrawn from treatment immediately.

6 INVESTIGATIONAL MEDICINAL PRODUCT AND PLACEBO

6.1 STUDY DRUG

6.1.1 Study Drug Identification

Gabapentin 300 mgs hard capsule

Placebo

6.1.2 Study Drug Manufacturer

Actavis UK Limited

6.1.3 Marketing Authorisation Holder

Actavis UK Limited
Whiddon Valley
Barnstaple
Devon EX32 8NS

6.1.4 Labelling and Packaging

Packaging and labelling of the over-encapsulated gabapentin and placebo will be carried out by:

Bilcare GCS (Europe) Ltd.
Waller House
Elvicta Business Park
Crickhowell Powys NP8 1DF
UK

6.1.5 Storage

The medicinal product should be stored at room temperature not above 25ºC

6.1.6 Summary of Product Characteristics or Investigators Brochure

The Summary of Product Characteristics (SmPC) is given in Appendix 1.

6.2 PLACEBO

The placebo will be manufactured, labelled and packed by Bilcare GCS (Europe) Ltd

6.3 DOSING REGIME

Participants will start on 300mg gabapentin daily and will increase in 300 mg increments each week until they report a 50% pain reduction, or side effects (eg dizziness, somnolence, mood changes, appetite and poor concentration), up to a maximum dose of 2700 mgs. Patients will be advised regarding their dosing regime weekly by a member of the research team who will phone until optimum dose is reached. It will be recommended that the drug should be taken in three equally divided doses daily. Participants will be advised to remain on the maximum tolerated dose for up to 6 months. The same protocol will be used for the placebo. When the participant stops treatment then then the dose will be tapered down over 7 - 10 days at the clinician's discretion.

6.4 DOSE CHANGES

See above. If side effects prevent escalation of the dose one week then participants will be advised to try and increase the drug the following week.

6.5 PARTICIPANT COMPLIANCE

All participants will be given a diary at randomisation and asked to document daily drug use (including self-medication and alternative treatments, eg acupuncture), side effects, reasons for drug escalation or reduction. We will also include specific healthcare resource use questions (e.g. attendance at GP, hospital). We will record the proportion of participants who completed their diaries with no missing data.

6.6 OVERDOSE

Acute, life-threatening toxicity has not been observed with gabapentin overdoses of up to 49g. Symptoms of overdose include dizziness, double vision, slurred speech, drowsiness, lethargy, and mild diarrhoea. All patients have recovered fully with supportive care. Reduced absorption of gabapentin at higher doses may limit drug absorption at the time of overdosing and, hence, minimize toxicity from overdoses.

Although gabapentin can be removed by haemodialysis, based on prior experience it is usually not required. However, in patients with severe renal impairment, haemodialysis may be indicated.

An oral lethal dose of gabapentin was not identified in mice and rats given doses as high as 8000 mg/kg. Signs of acute toxicity in animals included ataxia, laboured breathing, ptosis, hypoactivity, or excitation.

6.7 OTHER MEDICATIONS

6.7.1 Non-Investigational Medicinal Products

6.7.2 Permitted Medications

Participants may take the following concomitant medications during the course of the study, but those other than pain and anti-emetic medications must have been used at a stable dose for 30 days or more prior to randomisation.

Class of allowable (with restrictions) medication		Examples (not comprehensive list)	Allowable treatment dose
Medication used for pain relief	Analgesics	Oral paracetamol (acetaminophen)	Maximum dose of paracetamol or any paracetamol-containing products ≤4 g/day (8x500 mg/day)
	Anti-inflammatory	Aspirin<br><br>OTC NSAID ibuprofen, diclofenac, Stable inhaled corticosteroids	≤325 mg/day for prophylaxis use only (as antithrombotic agent).<br><br>Per product label
	Opiods	Oxycodone<br>Codeine<br>Dihydrocodeine<br>Morphine<br>Methadone<br>Tramadol<br>Fentanyl<br>Buprenorphine<br>Tapentadol	Per product label<br><br>Anyone given morphine will be carefully observed for signs of CNS depression, eg somnolence, and the dose of gabapentin or morphine will be reduced appropriately. Morphine will only be given on an inpatient basis.
	Non-harmacological therapies	Physical therapy, massage, chiropractic care, psychological care, exercise, yoga, medication	
	Local anaesthetics	Topical lidocaine	Per product label
Medication used for mood disorders	Anti-depressants	SSRIs including citalopram, fluoxetine, fluvoxamine, paroxetine, sertraline	Per product label
	Sedative	Midazolam/temazepam	Per product label
Medication used for control of nausea/vomiting	Anti-emetics	5HT-3 antagonists are strongly recommended for use at the start of surgery<br><br>H1 (dimenhydrinate)	Per product label

Medication used for insomnia	Hypnotics	Zopiclone	3.75-7.5 mgs/night. Intermittent use for insomnia only, no more than 3 doses a week.
		Zolpidem	5-10 mg/night. Intermittent use for insomnia only, no more than 3 doses a week.
		Zaleplon	5-10 mg/night. Intermittent use for insomnia only, no more than 3 doses a week
	Benzo-diazepines	Temazepam, triazolam	Intermittent use for insomnia only, no more than 3 doses a week.
	Antihistamines	Diphenhydramine, doxylamine	Intermittent use for insomnia only, no more than 3 doses a week.

6.7.3 Prohibited Medications

Prohibited from randomisation

Class of prohibited medications		Examples (not comprehensive list)	Minimum washout periods prior to study medication dosing
Anxiolytics, sedative hypnotics	Benzo-diazepines	Alprazolam, clonazepam, diazepam, flurazepam, lorazepam, quazepam, triazolam, temazepam (see table above for allowed and intermittent use for insomnia	≥7 days
Used for pain or may confound pain measurement	Barbiturates/ barbiturate combinations/ belladonna alkaloids	Amobarbital, butabarbital, metharbital, pentobarbital, phenobarital, secobarbital, atropine sulfate, hyoscyamine sulfate, scopolamine hydrobromide, antrocol (atropine and Phenobarbital) butibel (belladonna and butabarbital), donnatal (atropine sulfate, hyoscyamine sulfate, scopolamine hydrobromide and Phenobarbital)	≥7 days
	Antiepileptics	Levetiracetam, carbamazepine, clonazepam, pregabalin, lamotrigine, phenytoin, topiramate and valproic acid	≥7 days
	Oral/IV/ per-rectum corticosteroids	Dexamethasone, hydrocortisone, methylprednisolone, prednisolone, triamicinolone	≥7 days
Medication used for control of nausea/vomiting	Anti-emetics	Corticosteroids and neuroleptic anti-emetics excluding metaclopramide.<br>See above table for allowable	≥7 days
Potential retinotoxins	Potential retinotoxins	Hydroxychloroquine, deferoxamine, thioridazine, vigabatrin	Participants who have ever take these are not eligible for the study
Others	Monoamine oxidase inhibitors (MAOIs)	Phenelzine, tranycypromine, moclobemide, socarboxazid	MAOIs ≥21 days

	Other psychoactive drugs with psychoactive effects	Amphetamines, cannabis, cocaine, dimethyltryptamine (DMT), dimenthoxymethylamphetamine (DOM or "STP"), heroin, LSD, Ecstasy, PCP (phencyclidine), psilocybin	≥14 days
	Herbal psychoactive and miscellaneous supplement treatments	St John's Wort, α-lipoic acid, chromium picolinate, evening primrose oil, fatty acid supplements, myoinositol	≥14 days
	Peripherally acting antiadrenergic	Guanethidine monosulfate	≥3 months
		Sympathetic blocks, acupuncture, botox, any other non-pharmacological treatment involving needle	≥3 months
	Electro-analgesia/ electrotherapy	Transcutaneous electrical nerve stimulation (TENS)	Prohibited during the study.

7 STUDY ASSESSMENTS

7.1 SAFETY ASSESSMENTS

- Medical history
- Pregnancy test
- Prior and concomitant medications
- Adverse events

7.2 STUDY ASSESSMENTS (See Appendix 5 for additional detail)

Baseline (0 months):	**Visit 1**<br>Informed consent<br>Screening<br>Eligibility confirmed<br>Randomisation to gabapentin or placebo<br>Treatment diary<br>Questionnaire
Week 1	Telephone interview
Week 2	Telephone interview (if dose escalation not finished)
Week 3	Telephone interview (if dose escalation not finished)
Week 4	Telephone interview (if dose escalation not finished)
Week 5	Telephone interview (if dose escalation not finished)
Week 6 (+/- 7 days)	**Visit 2**<br>Short interview<br>Dispensing of drugs if necessary

3 months (+/- 7 days)	**Visit 3**	
	Questionnaire	
	Dispensing of drugs if necessary	
	Brain fMRI (Edinburgh subset)	
6 months(+/- 7 days)	**Visit 4**	
	Questionnaire	
	Unblinding	
	Collection of treatment diary	
	Collection of any unused study medications	
3 - 6 months	Focus groups (subset)	

The following measures will be completed by the subject in a daily treatment diary beginning on Day 1 of treatment.

- Time and dose of gabapentin taken
- Reason for any change in trial medication dose
- Concomitant medications taken
- Alternative therapies used
- Any visits to a healthcare professional
- Adverse events
- Weekly average Lickert pain score

fMRI

24 participants in Edinburgh will be allocated to undergo functional magnetic resonance imaging (fMRI) of the brain at 3 months (as long as they satisfy safety issues related to the procedure). The study of fMRI requires the need to contrast periods of brain activation (induced by pain) with periods of rest. Therefore, subjects will be asked to lie still and a weighted ball will be applied to the abdomen at regular intervals during the procedure (block design with alternating periods of 16 seconds of 'pain' and 16 seconds of 'rest') (Liu et al, 2001). Pain levels will vary per subjects so we will include a 'rating scale' to allow us to do a regression analysis of fMRI level of activation as a function of the level of pain. Pain levels also vary according to the woman's menstrual cycle (reference). We will carry out the fMRI at the same stage of each participant's cycle. We will take a blood sample to measure hormone levels. Each fMRI will last ~30 mins: structural scans T1 and T2 (~10 mins), fMRI scan 16 seconds on/off repeated 20 times (~10 mins) and time for subject to get into and out of scanner and apply weighted ball in position (~10 mins). Discontinuation rules in case of patient distress will be as follows. Before the fMRI, we will assess the level of discomfort associated with the 'ball test' (plus obtain the 'rating'). We will also use a thermal resistor to deliver noxious thermal stimulation of a pre-determined intensity (the temperature adjusted for each subject to produce a rating of this intensity) as has been successfully used previously (VINCENT K, et al. Pain 2011 152(9):1966-75). Participants will also be given the opportunity to withdraw from this component of the study pre-fMRI. In addition, in the scanners, there is a button that participant can press to stop the scan. This will allow participants to withdraw from this component of the study during the fMRI. The fMRI studies will be performed in Edinburgh (3T Siemens MRI), supported by the Scottish Imaging Network, SINAPSE. We will calibrate the fMRI protocols by asking up to 4 healthy volunteers to undergo this procedure. They will complete a CRIC consent and safety form before taking part.

8 DATA COLLECTION

Screening: A member of the research team will carry out a screening visit to assess eligibility. All data will be recorded on a CRF and transferred to a secure database. Participants will be reminded to use effective contraception for the duration of the study and for one week after their last dose of gabapentin.

Participant Log: The clinical research team will keep an electronic log of women who fulfil the eligibility criteria, women who are invited to participate in the study, women recruited and women who leave the trial early. Reasons for non-recruitment (eg non-eligibility, refusal to participate, administrative error) will also be recorded. We will attempt to collect reasons for non-participation from women who decline to take part after previously providing contact details. During the course of the study, we will document reasons for withdrawal from the study and loss to follow-up.

Treatment diaries: details are documented in Section 6.5. All medications taken after screening and any medication other than the trial treatment taken during the study will be recorded in the treatment diary. This includes prescription and non-prescription treatment such as contraceptives, vitamins, topical preparations, herbal preparations and non-pharmacological therapies. Permissible and excluded concomitant medications are itemised. These restrictions apply during the period from screening to end of treatment. The permissible supplemental pain medications may be initiated at any time during the study (unless specified otherwise).

Questionnaires: A questionnaire will be given to all participants at randomisation (0 months) and at 3 months. This will include questions to capture the baseline demographic and clinical characteristics of the participants and include the following validated tools:

1. Neuropathic Pain Questionnaire (NPQ) (including Lickert pain score)
2. Brief Pain Inventory (BPI)
3. Pain Disability Questionnaire (PDQ)
4. Hospital Anxiety and Depression Score (HADS)
5. EQ5D QoL
6. WHO QoL
7. MYMOP patient-generated outcome questionnaire

A further questionnaire will also be given to all participants at 6 months which will include the above and additional questions on whether they believed they were receiving Gabapentin or placebo, and also questions on acceptability of the allocation medication (and compliance) and on the acceptability of the above data collection methods. Lastly, we will ask the participants to complete a brief anonymous questionnaire once they have submitted their treatment diaries to assess level of adherence to diary keeping.

Focus groups: A purposive sample (based on age, social class and severity of symptoms) of 10 Edinburgh women (including some undergoing fMRI) and 10 Aberdeen women will be invited to participate in focus group discussions of the trial experience. Women who do not wish to participate in a focus group will be offered individual interviews using the same interview schedule. This will enable important issues arising in the focus groups to be explored in greater depth. Up to 20 interviews will be performed. Group/individual interviews will be audio-recorded, transcribed verbatim and analysed thematically to identify the issues of importance to participants.

9 STATISTICS AND DATA ANALYSIS

9.1 SAMPLE SIZE CALCULATION

We have used a confidence interval (CI) approach (Thabane 2010) to estimate the sample size to establish feasibility based on a loss to follow-up of <20%. A 95% CI for 20% of 60 patients (12/60) is 11% to 32%. We estimate that we will recruit ~3-4 patients per month from each centre and aim to recruit 60 patients over a nine-month recruitment period. Each centre performs 6-7 laparoscopies per month that fit the inclusion criteria. A power calculation for the fMRI sub-studies is more difficult due to the complexity of the model (Mumford and Nichols 2008). The signal noise ratio changes in space and therefore power depends on the effect size that differs from region to region. We have estimated that we will need to recruit 24 women to determine where there are differences using fixed effect analyses

9.2 PROPOSED ANALYSES

(i) Determine recruitment and retention rates in two centres

Using the information collected from the participant log, we will determine the number of patients recruited from the pool of eligible women and a >50% recruitment will be deemed acceptable. While a retention rate of 100% would be ideal, we will consider a rate of 90% satisfactory. We will provide an estimate of the proportion and its 95% confidence interval. In addition, we will determine the nature and number of unanswered questions in each questionnaire and identify reasons for non-response through the focus groups and participant interviews in order to optimise data collection in the future trial.

(ii) Effectiveness and acceptability of proposed methods of recruitment, randomisation, drug treatments, questionnaires, use of brain fMRI and follow-up?

These areas will be explored in the focus group discussions and assessed quantitatively using additional questions included in participant questionnaires administered at 6 months. Due to the conflicting literature about the benefits of methods such as prescription monitoring, pill counting and devices for monitoring the self-administration of medicines (Melnikow and Kiefe, 1994), data on blinding and compliance to treatment will be derived from questionnaires at 6 months. We aim to determine if treatment is acceptable in terms of self-reported compliance (from treatment diaries). Although this is a pilot study and the sample size is small, we will assess the effect of any non-compliance on the LICKERT score by performing protocol and intention to treat analyses. This information along with health professionals' and clinical research nurses' views (as assessed by questionnaire) will be used to inform the design of the future RCT. (iii) Sensitivity of brain fMRI to study the effect of gabapentin in the management of CPP

The fMRI data will be analysed using a voxelwise approach and hierarchical linear modelling. An ANOVA [pre/post treatment*gabapentin/ placebo] will be performed to investigate which areas of the brain gabapentin acts on compared to placebo. A regression analysis between brain activity and the behavioural measures will also be performed to evaluate the relationship between gabapentin brain activity and pain reduction. The brain fMRI scan results will then be compared with other studies of gabapentin (Iannetti 2005, Governo 2008) to determine the sensitivity of this approach to study the mechanism of action of gabapentin in CPP. We will use published fMRI methodology (Mumford & Nichols 2008) informed by the pilot to power future fMRI studies.

(iv) Pre-trial economic model and value of information analysis

In addition to data relating to the clinical and quality of life parameters, data on healthcare resource use will also be collected. A decision model will be developed to estimate the costs from the perspective of the NHS, and health outcomes in terms of quality

of life and quality adjusted life years associated with gabapentin and placebo based on the data from this pilot study and the literature. A probabilistic decision model will be constructed to simulate the clinical pathways associated with gabapentin and placebo, according to the guidance set out by NICE (NICE 2008). The basic model structure will consist of two arms, replicating the clinical consequences of patients receiving gabapentin and placebo. The main data source relating to the key parameters of the model will be provided by the pilot study. The mean costs and quality adjusted life years associated with both arms will be calculated for the modelling period (duration of the trial). Cost utility analysis will be carried out and incremental cost per quality-adjusted life years gained will be calculated. Particular consideration will be given to the potential for cost effectiveness to vary by particular patient characteristics or risk groups where suggested by the literature. Probability sensitivity analysis will be used to characterise uncertainty in parameters of the model, and presented using cost-effectiveness acceptability curves. Standard univariate sensitivity analysis will be carried out to explore areas of structural uncertainty in the analysis. Finally, a value of information analysis on the expected value of perfect information will also be carried out to quantify potential value of further research based on the difference between expected net benefit with perfect information and existing information.

10 ADVERSE EVENTS

The Investigator is responsible for the detection and documentation of events meeting the criteria and definitions detailed below.

Participants will collect information about adverse events in their treatment diaries. However, they will be instructed to contact the clinical research team at any time after consenting to join the trial is they have an event that requires hospitalisation, or an event that results in persistent or significant disability or incapacity. The participants will be contacted weekly by a member of the research staff until they have reached their optimal dosage. Gabapentin is generally well tolerated in the management of other chronic pain conditions and SAEs are not anticipated. Any SAEs that occur after joining the trial will be reported in detail in the participant's medical notes and followed up until resolution of the event.

Full details of contraindications and side effects that have been reported following administration of the IMP can be found in the relevant Summary of Product Characteristics (SmPC).

Participants will be instructed to contact their Investigator at any time after consenting to join the trial if any symptoms develop. All adverse events (AE) that occur after joining the trial will be reported in detail in the Case Report Form (CRF) or AE form. In the case of an AE, the Investigator will ensure that the appropriate treatment is instigated by the appropriate medical personnel. Participants with AEs present at the last visit must be followed up until resolution of the event.

10.1 DEFINITIONS

An adverse event (AE) is any untoward medical occurrence in a clinical trial participant which does not necessarily have a causal relationship with an investigational medicinal product (IMP).

An adverse reaction (AR) is any untoward or unintended response to an IMP which is related to any dose administered to that participant.

An unexpected adverse reaction (UAR) is an adverse reaction that is not consistent with the applicable product information for the IMP, e.g. the SmPC for a licensed product.

A serious adverse event (SAE), serious adverse reaction (SAR) or suspected unexpected serious adverse reaction (SUSAR) is any AE, AR or UAR that at any dose:

- results in death;
- is life threatening* (i.e. the participant was at risk of death at the time of the event; it does not refer to an event which hypothetically might have caused death if it were more severe);
- requires in-patient hospitalisation^ or prolongation of existing hospitalisation;
- results in persistent or significant disability or incapacity;
- is a congenital anomaly or birth defect.

^Any hospitalisation that was planned prior to randomisation will not meet SAE criteria. Any hospitalisation that is planned post randomisation will meet the SAE criteria.

10.2 DETECTING AEs AND SAEs

All AEs and SAEs will be recorded from the time a participant signs the consent form to take part in the study until end of treatment.

Participants will be asked to record the occurrence of AEs/SAEs in their treatment diaries during the study. They will not be asked to document the occurrence of any common ailments not associated with the drug eg colds and coughs. Open-ended and non-leading verbal questioning of the participant will be used to enquire about AE/SAE occurrence. Participants will also be asked if they have been admitted to hospital, had any accidents, used any new medicines or changed concomitant medication regimens. If there is any doubt as to whether a clinical observation is an AE, the event will be recorded. Participants will also be asked to record occurrence of any AEs in their daily treatment diaries. The participant's mood will be monitored by the research team using open ended questions during phone consultations and through the use of questionnaires at 3 and 6 months eg Hospital Anxiety and Depression Scale.

AEs and SAEs may also be identified by support departments e.g. laboratories.

10.3 RECORDING AEs AND SAEs

When an AE/SAE occurs, it is the responsibility of the Investigator to review all documentation (e.g. hospital notes, laboratory and diagnostic reports) related to the event. The Investigator will then record all relevant information in the CRF and on the SAE form (if the AE meets the criteria of serious).

Information to be collected includes dose, type of event, onset date, Investigator assessment of severity and causality, date of resolution as well as treatment required, investigations needed and outcome.

10.4 ASSESSMENT OF AEs AND SAEs

Seriousness, causality, severity and expectedness will be assessed as though the participant is taking active IMP. Cases that are considered serious and possibly related to IMP and unexpected (i.e. SUSARs) will be unblinded.

The Investigator is responsible for assessing each AE.

The Chief Investigator (CI) may not downgrade an event that has been assessed by an Investigator as an SAE or SUSAR, but can upgrade an AE to an SAE, SAR or SUSAR if appropriate.

10.4.1 Assessment of Seriousness

The Investigator will make an assessment of seriousness as defined in Section 10.1.

10.4.2 Assessment of Causality

The Investigator will make an assessment of whether the AE/SAE is likely to be related to the IMP according to the definitions below.

All AEs/SAEs judged as having a reasonable suspected causal relationship (ie possibly related) to the IMP will be considered as related to the IMP (ARs/SARs).

Where non Investigational Medicinal Products (NIMPs) e.g. rescue/escape drugs are given: if the AE is considered to be related to an interaction between the IMP and the NIMP, or where the AE might be linked to either the IMP or the NIMP but cannot be clearly attributed to either one of these, the event will be considered as an AR/SAR.

Unrelated: where an event is not considered to be related to the IMP.

Possibly Related: Possibly Related: The nature of the event, the underlying medical condition, concomitant medication or temporal relationship make it possible that the AE has a causal relationship to the study drug.

Alternative causes such as natural history of the underlying disease, other risk factors and the temporal relationship of the event to the treatment should be considered and investigated. The blind should not be broken for the purpose of making this assessment.

10.4.3 Assessment of Severity

The Investigator will make an assessment of severity for each AE/SAE and record this on the CRF or AE form according to one of the following categories:

Mild: an event that is easily tolerated by the participant, causing minimal discomfort and not interfering with every day activities.

Moderate: an event that is sufficiently discomforting to interfere with normal everyday activities.

Severe: an event that prevents normal everyday activities.

Note: the term 'severe', used to describe the intensity, should not be confused with 'serious' which is a regulatory definition based on participant/event outcome or action criteria. For example, a headache may be severe but not serious, while a minor stroke is serious but may not be severe.

10.4.4 Assessment of Expectedness

If an event is judged to be an AR/SAR, the evaluation of expectedness will be made based on knowledge of the reaction and the relevant product information documented in the SmPC.

The event may be classed as either:

Expected: the AR is consistent with the toxicity of the IMP listed in the SmPC.

Unexpected: the AR is not consistent with the toxicity in the SmPC.

10.5 REPORTING OF SAEs/SARs/SUSARs

Once the Investigator becomes aware that an SAE has occurred in a study participant, the information will be reported to the ACCORD Research Governance and QA Office immediately or within 24 hours. If the Investigator does not have all information regarding an SAE, they should not wait for this additional information before notifying ACCORD. The SAE report form can be updated when the additional information is received.

The SAE report will provide an assessment of causality and expectedness at the time of the initial report to ACCORD according to Sections 10.4.2, Assessment of Causality and 10.4.4, Assessment of Expectedness.

The SAE form will be transmitted by fax to ACCORD on +44 (0)131 242 9447 or may be transmitted by hand to the office.

All reports faxed to ACCORD and any follow up information will be retained by the Investigator in the Investigator Site File (ISF).

10.6 REGULATORY REPORTING REQUIREMENTS

The ACCORD Research Governance & QA Office is responsible for pharmacovigilance reporting on behalf of the co-sponsors (Edinburgh University and NHS Lothian).

The ACCORD Research Governance & QA Office has a legal responsibility to notify the regulatory competent authority and relevant ethics committee (Research Ethics Committee (REC) that approved the trial). Fatal or life threatening SUSARs will be reported no later than 7 calendar days and all other SUSARs will be reported no later than 15 calendar days after ACCORD is first aware of the reaction.

ACCORD will inform Investigators at participating sites of all SUSARs and any other arising safety information.

A Development Safety Update Report (DSUR) will be submitted to the regulatory competent authority and main REC listing all SARs and SUSARs.

10.7 FOLLOW UP PROCEDURES

After initially recording an AE or recording and reporting an SAE, the Investigator will follow each participant until resolution or death of the participant. Follow up information on an SAE will be reported to the ACCORD office.

AEs still present in participants at the last study visit will be monitored until resolution of the event or until no longer medically indicated.

11 PREGNANCY

Pregnancy is not considered an AE or SAE; however, the Investigator will collect pregnancy information for any female participants who become pregnant while participating in the study. The Investigator will record the information on a Pregnancy Notification Form and submit this to the ACCORD office within 14 days of being made aware of the pregnancy.

All pregnant female participants will be followed up until following the outcome of the pregnancy.

Participants will be asked not to try and get pregnant for the duration of the study. Use of contraception will be recorded at screening.

12 TRIAL MANAGEMENT AND OVERSIGHT ARRANGEMENTS

12.1 TRIAL MANAGEMENT GROUP

The trial will be coordinated by a Project Management Group, consisting of the grant holders (Chief Investigator and Principal Investigator in Aberdeen), the Trial Manager and coordinating nurse.

The Trial Manager will oversee the study and will be accountable to the Chief Investigator. The Trial Manager will be responsible for checking the CRFs for completeness, plausibility and consistency. Any queries will be resolved by the Investigator or delegated member of the trial team.

A Delegation Log will be prepared for each site, detailing the responsibilities of each member of staff working on the trial.

12.2 TRIAL STEERING COMMITTEE

A Trial Steering Committee (TSC) will be established to oversee the conduct and progress of the trial. The terms of reference of the Trial Steering Committee, the draft template for reporting and the names and contact details are detailed in Appendix 2. The TSC will meet before study starts and will meet at least once during the study. They will be sent updates on the progress of the study.

12.3 DATA MONITORING COMMITTEE

An independent Data Monitoring Committee (DMC) will be established to oversee the safety of participants in the trial. The terms of reference of the Data Monitoring Committee and the names and contact details are detailed in Appendix 3. The DMC will meet before the study starts and will meet at least once during the study. They will be sent updates on progress of the study every 6 months.

12.4 INSPECTION OF RECORDS

Investigators and institutions involved in the study will permit trial related monitoring and audits on behalf of the sponsor, REC review, and regulatory inspection(s). In the event of an audit or monitoring visit, the Investigator agrees to allow the representatives of the sponsor direct access to all study records and source documentation. In the event of regulatory inspection, the Investigator agrees to allow inspectors direct access to all study records and source documentation.

12.5 RISK ASSESSMENT

An independent risk assessment will be performed by an ACCORD Clinical Trials Monitor to determine if monitoring is required and if so, at what level. An independent risk assessment will also be carried out by the ACCORD Quality Assurance Group to determine if an audit should be performed before/during/after the study and if so, at what locations and at what frequency.

12.6 STUDY MONITORING AND AUDIT

An ACCORD Clinical Trials Monitor or an appointed monitor will visit the Investigator site prior to the start of the study and during the course of the study if required, in accordance with the monitoring plan. Investigator sites will be risk assessed by the ACCORD QA Manager, or designee, in order to determine if audit by the ACCORD QA group is required.

13 GOOD CLINICAL PRACTICE

13.1 ETHICAL CONDUCT

The study will be conducted in accordance with the principles of the International Conference on Harmonisation Tripartite Guideline for Good Clinical Practice (ICH GCP).

A favorable ethical opinion will be obtained from the appropriate REC and local R&D approval will be obtained prior to commencement of the study.

13.2 REGULATORY COMPLIANCE

The study will not commence until a Clinical Trial Authorisation (CTA) is obtained from the appropriate Regulatory Authority. The protocol and study conduct will comply with the Medicines for Human Use (Clinical Trials) Regulations 2004, and any relevant amendments.

13.3 INVESTIGATOR RESPONSIBILITIES

The Investigator is responsible for the overall conduct of the study at the site and compliance with the protocol and any protocol amendments. In accordance with the principles of ICH GCP, the following areas listed in this section are also the responsibility of the Investigator. Responsibilities may be delegated to an appropriate member of study site staff.

13.3.1Informed Consent

The Investigator is responsible for ensuring informed consent is obtained before any protocol specific procedures are carried out. The decision of a participant to participate in clinical research is voluntary and should be based on a clear understanding of what is involved.

Participants must receive adequate oral and written information – appropriate Participant Information and Informed Consent Forms will be provided. The oral explanation to the participant will be performed by the Investigator or qualified delegated person, and must cover all the elements specified in the Participant Information Sheet and Consent Form.

The participant must be given every opportunity to clarify any points they do not understand and, if necessary, ask for more information. The participant must be given sufficient time to consider the information provided. It should be emphasised that the participant may withdraw their consent to participate at any time without loss of benefits to which they otherwise would be entitled.

The participant will be informed and agree to their medical records being inspected by regulatory authorities and representatives of the sponsor(s) but understand that their name will not be disclosed outside the hospital.

The Investigator or delegated member of the trial team and the participant will sign and date the Informed Consent Form(s) to confirm that consent has been obtained. The participant will receive a copy of this document and a copy filed in the Investigator Site File (ISF) and participant's medical notes.

13.3.2 Study Site Staff

The Investigator must be familiar with the IMP, protocol and the study requirements. It is the Investigator's responsibility to ensure that all staff assisting with the study are adequately informed about the IMP, protocol and their trial related duties.

13.3.3 Data Recording

The Principle Investigator is responsible for the quality of the data recorded in the CRF at each Investigator Site.

13.3.4 Investigator Documentation

Prior to beginning the study, each Investigator will be asked to provide particular essential documents to the ACCORD Research Governance & QA Office, including but not limited to:

- An original signed Investigator's Declaration (as part of the Clinical Trial Agreement documents);
- Curriculum vitae (CV) signed and dated by the Investigator indicating that it is accurate and current.

The ACCORD Research Governance & QA Office will ensure all other documents required by ICH GCP are retained in a Trial Master File (TMF), where required, and that appropriate documentation is available in local ISFs.

13.3.5 GCP Training

All study staff must hold evidence of appropriate GCP training.

13.3.6 Confidentiality

All laboratory specimens, evaluation forms, reports, and other records must be identified in a manner designed to maintain participant confidentiality. All records must be kept in a secure storage area with limited access. Clinical information will not be released without the written permission of the participant. The Investigator and study site staff involved with this study may not disclose or use for any purpose other than performance of the study, any data, record, or other unpublished, confidential information disclosed to those individuals for the purpose of the study. Prior written agreement from the sponsor or its designee must be obtained for the disclosure of any said confidential information to other parties.

13.3.7 Data Protection

All Investigators and study site staff involved with this study must comply with the requirements of the Data Protection Act 1998 with regard to the collection, storage, processing and disclosure of personal information and will uphold the Act's core principles. Access to collated participant data will be restricted to those clinicians treating the participants.

Computers used to collate the data will have limited access measures via user names and passwords.

Published results will not contain any personal data that could allow identification of individual participants.

14 STUDY CONDUCT RESPONSIBILITIES

14.1 PROTOCOL AMENDMENTS

Any changes in research activity, except those necessary to remove an apparent, immediate hazard to the participant in the case of an urgent safety measure, must be reviewed and approved by the Chief Investigator.

Amendments to the protocol must be submitted in writing to the appropriate REC, Regulatory Authority and local R&D for approval prior to participants being enrolled into an amended protocol.

14.2 PROTOCOL VIOLATIONS AND DEVIATIONS

Investigators will not implement any deviation from the protocol without agreement from the Chief Investigator and appropriate REC, Regulatory Authority and R&D approval except where necessary to eliminate an immediate hazard to trial participants.

In the event that an Investigator needs to deviate from the protocol, the nature of and reasons for the deviation will be recorded in the CRF. If this necessitates a subsequent protocol amendment, this should be submitted to the REC, Regulatory Authority and local R&D for review and approval if appropriate.

14.3 SERIOUS BREACH REQUIREMENTS

A serious breach is a breach which is likely to effect to a significant degree:

(a) the safety or physical or mental integrity of the participants of the trial; or

b) the scientific value of the trial.

If a potential serious breach is identified by the Chief investigator, Principal Investigator or delegates, the co-sponsors (accord. seriousbreach@ed.ac.uk) must be notified within 24 hours. It is the responsibility of the co-sponsors to assess the impact of the breach on the scientific value of the trial, to determine whether the incident constitutes a serious breach and take the appropriate action.

Not every violation from the protocol needs to be reported to the regulatory authority as a serious breach. If the sponsor(s) deem the incident to be a violation that does not constitute a serious breach from the protocol when identified, corrective and preventative actions will be taken where appropriate and they will be recorded in file notes, held within the TMF and ISF.

14.4 STUDY RECORD RETENTION

All study documentation will be kept for a minimum of 5 years from the protocol defined end of study point. When the minimum retention period has elapsed, study documentation will not be destroyed without permission from the sponsor.

14.5 END OF STUDY

The end of study is defined as the last participant's end of treatment.

The Investigators and/or the trial steering committee and/or the co-sponsor(s) have the right at any time to terminate the study for clinical or administrative reasons.

The end of the study will be reported to the REC and Regulatory Authority within 90 days, or 15 days if the study is terminated prematurely. The Investigators will inform participants of the premature study closure and ensure that the appropriate follow up is arranged for all participants involved.

A summary report of the study will be provided to the REC and Regulatory Authority within 1 year of the end of the study.

14.6 CONTINUATION OF DRUG FOLLOWING THE END OF STUDY

Following unblinding, participants who have been taking gabapentin and had benefit from it will be offered the chance to continue the gabapentin in consultation with their consultant and GP.

14.7 INSURANCE AND INDEMNITY

The co-sponsors are responsible for ensuring proper provision has been made for insurance or indemnity to cover their liability and the liability of the Chief Investigator and staff.

The following arrangements are in place to fulfil the co-sponsors' responsibilities:

- The Protocol has been designed by the Chief Investigator and researchers employed by the University of Edinburgh and collaborators. The University has insurance in place (which includes no-fault compensation) for negligent harm caused by poor protocol design by the Chief Investigator and researchers employed by the University.
- Sites participating in the study will be liable for clinical negligence and other negligent harm to individuals taking part in the study and covered by the duty of care owed to them by the sites concerned. The co-sponsors require individual sites participating in the study to arrange for their own insurance or indemnity in respect of these liabilities.
- Sites which are part of the United Kingdom's Nation Health Service will have the benefit of NHS Indemnity.

- Sites outwith the United Kingdom will be responsible for arranging their own indemnity or insurance for their participation in the study, as well as for compliance with local law applicable to their participation in the study.
- The manufacturer supplying IMP has accepted limited liability related to the manufacturing and original packaging of the study drug and to the losses, damages, claims or liabilities incurred by study participants based on known or unknown Adverse Events which arise out of the manufacturing and original packaging of the study drug, but not where there is any modification to the study drug (including without limitation re-packaging and blinding).

15 REPORTING, PUBLICATIONS AND NOTIFICATION OF RESULTS

15.1 AUTHORSHIP POLICY

Ownership of the data arising from this study resides with the study team. On completion of the study, the study data will be analysed and tabulated, and a clinical study report will be prepared in accordance with ICH guidelines.

15.2 PUBLICATION

The clinical study report will be used for publication and presentation at scientific meetings. Investigators have the right to publish orally or in writing the results of the study.

Summaries of results will also be made available to Investigators for dissemination within their clinics (where appropriate and according to their discretion).

15.3 PEER REVIEW

The protocol has undergone external peer review as part of the application process to Chief Scientist Office (Scotland) for funding.

16 REFERENCES

CHEONG Y, *et al*. Best Pract Res Clin Obstet Gynaecol. 2006 20(5):695-711.

DANIELS JP, KHAN KS. BMJ. 2010 341:c4834.

GOVERNO RJ, *et al*. Br J Pharmacol 2008 153(7):1558.

IANNETTI GD, *et al*. Proc Natl Acad Sci U S A 2005 102(50):18195.

KELLY AM. Emerg Med J. 2001 18(3):205-7.

LATTHE P, *et al*. BMC Public Health 2006 6:177.

LIU *et al*. Neuroimage 2001 13(4):759-73.

MELNIKOW J, KIEFE C. J Gen Intern Med. 1994 9(2):96-105.

MUMFORD JA, NICHOLS TE. Neuroimage 2008 39(1):261-8.

NICE (National Institute for Health and Clinical Excellence) 2008. Available online at: http://www.nice.org.uk/aboutnice/media/B52/A7/TAMethodsGuideUpdatedJune2008.pdf

SATOR-KATZENSCHLAGER SM, et al. Wien Klin Wochenschr 2005 117(21-22):761.

SERPELL MG *et al*. Pain. 2002 99(3):557-66.

STONES RW, MOUNTFIELD J. Cochrane Database Syst Rev 2000;(4):CD000387.

THABANE L, *et al*. BMC Med Res Methodol 2010 10:1.

VINCENT K, *et al*. BJOG 2009 116(2):240.

WIFFEN PJ, *et al*. Cochrane Database Syst Rev 2005 20;(3):CD005452.

APPENDIX 1: Summary of Product Characteristics

The manufacturer may change the SmPC, for this study as new information becomes available. The study will therefore adopt the manufacturer's current SmPC. The study team will monitor and review changes to the SmPC and consider the impact on the study and revise documents if required.

The SmPC is published on the electronic Medicines Compendium (eMC) showing the date it was published and the reasons for change.

At the time of writing this protocol the SmPC was last updated for Gabapentin 300 mgs on the eMC website on 23/06/2011, and is available at:

http://www.medicines.org.uk/EMC/medicine/24646/SPC/Gabapentin+300mg+Capsules/

APPENDIX 2: Trial Steering Committee

Chair:

Prof Jenny Higham
Deputy Principal of the Faculty of Medicine
Imperial College London
Tel: 020 759 49802
Email: jenny.higham@imperial.ac.uk

Members:

Dr Andrew Horne
MRC Centre for Reproductive Health
The Queen's Medical Research Institute
47 Little France Crescent
Edinburgh EH16 4TJ
Tel: 0131 242 6609
Email: Andrew.horne@ed.ac.uk

Professor Siladitya Bhattacharya
Division of Aberdeen Health Sciences
University of Aberdeen
Aberdeen Maternity Hospital
Aberdeen
Tel: 01224 550590
Email: s.bhattacharya@abdn.ac.uk

Ann Doust
University of Edinburgh
Room S7128
Simpson Centre for Reproductive Biology
Royal Infirmary of Edinburgh
51 Little France Crescent
Edinburgh EH16 4SA
Tel: 0131 242 9492
Email: ann.doust@ed.ac.uk

Prof Hilary Critchley
MRC Centre for Reproductive Health
The Queen's Medical Research Institute
47 Little France Crescent
Edinburgh EH16 4TJ
Tel: 0131 242 6439
Email: Hilary.Critchley@ed.ac.uk

Dr Patrick Chien
Obstetrics and Gynaecology
Ninewells Hospital Dundee
Email: patrick.chien@nhs.net

Steff Lewis
Centre for Population Health Sciences
University of Edinburgh Medical School
Teviot Place
Edinburgh H8 9AG
Tel: 0131 650 3198
Email: Steff.Lewis@ed.ac.uk

Dr Cyril Pernet
SBIRC
Division of Clinical Neurosciences
Western General Hospital
Crewe Road South
EH4 2XU
Tel: 0131 537 3661
Email: cyril.pernet@ed.ac.uk

Dr Maureen Porter
Division of Applied Health Sciences
University of Aberdeen
Aberdeen Maternity Hospital
Aberdeen Tel: 01224 554875
Fax:
Email: m.a.porter@abdn.ac.uk

Dr Olivia Wu
Centre for Population and Health Sciences
University of Glasgow
1 Lilybank Gardens
Glasgow G12 8RZ
Tel: 0141 330 3296
Fax: 0141 330 5018
Email: Olivia.wu@glasgow.ac.uk

Dr Stuart Jack
Department of Gynaecology
Aberdeen Royal Infirmary
Aberdeen AB25 2ZN
Tel: 01224 553471
Fax:
Email: sjackhome@btinternet.com

Dr John Wilson
Dept of Anaesthesia, Critical Care and Pain Medicine
Royal Infirmary of Edinburgh
Little France Crescent
Edinburgh EH16 4SA
Tel: 0131 242 3192
Fax:
Email: john.wilson@luht.scot.nhs.uk

APPENDIX 3: Data Monitoring Committee

Prof Khalid Khan (Queen Mary University of London)

Prof Richard Anderson (University of Edinburgh)

Mr Graeme MacLennan (University of Aberdeen)

APPENDIX 4: WORLD MEDICAL ASSOCIATION DECLARATION OF HELSINKI

Ethical Principles for Medical Research Involving Human Subjects

Adopted by the 18th WMA General Assembly, Helsinki, Finland, June 1964, and amended by the:

29th WMA General Assembly, Tokyo, Japan, October 1975

35th WMA General Assembly, Venice, Italy, October 1983

41st WMA General Assembly, Hong Kong, September 1989

48th WMA General Assembly, Somerset West, Republic of South Africa, October 1996

52nd WMA General Assembly, Edinburgh, Scotland, October 2000

53rd WMA General Assembly, Washington 2002 (Note of Clarification on paragraph 29 added)

55th WMA General Assembly, Tokyo 2004 (Note of Clarification on Paragraph 30 added)

59th WMA General Assembly, Seoul, October 2008

http://www.wma.net/e/policy/pdf/17c.pdf

Appendix 1: Flowchart - Pilot study (gabapentin for pelvic pain)

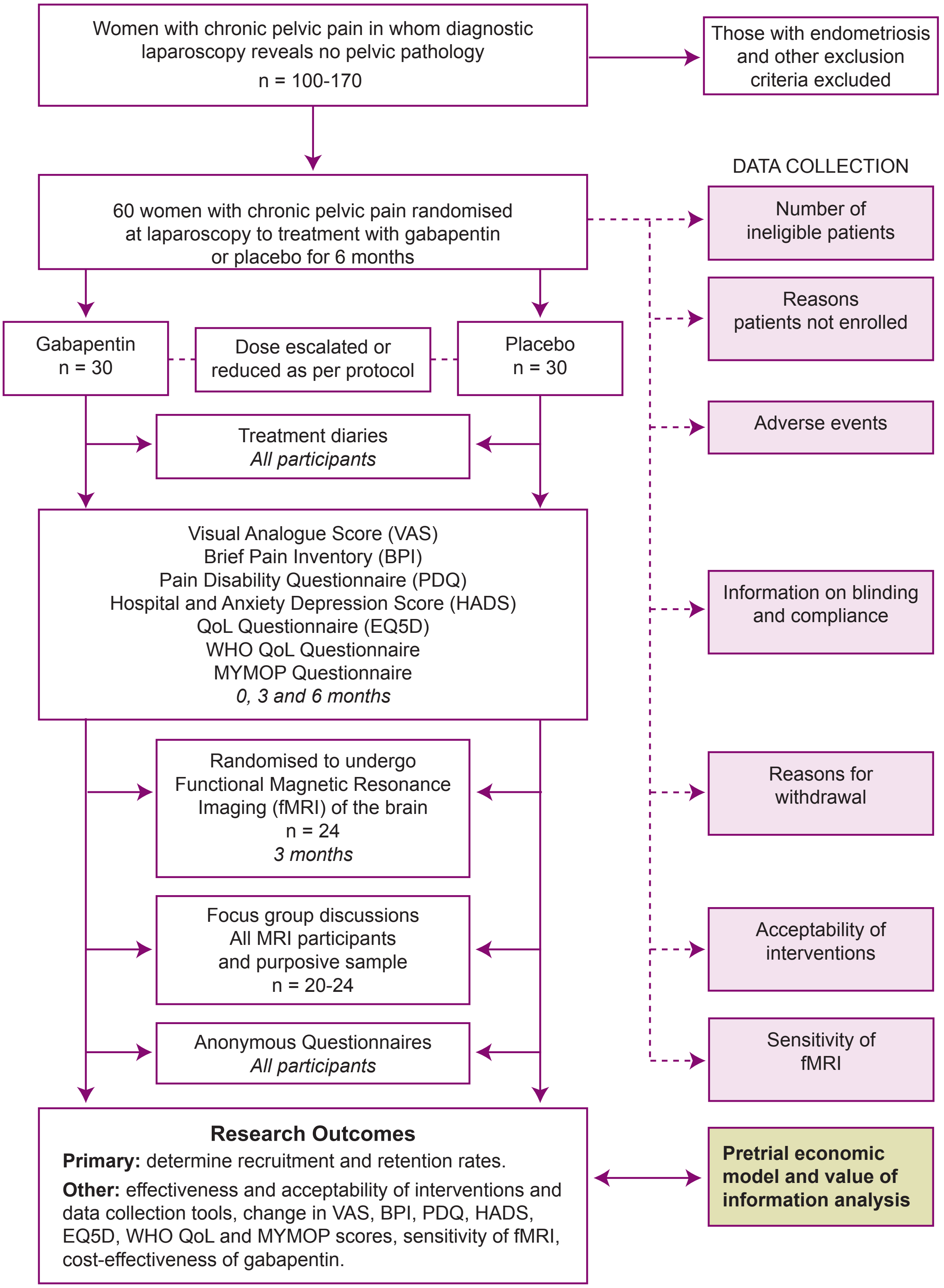

APPENDIX 6: STUDY FLOWCHART

Possible recruit identified by clinician
- Pelvic pain for >6 months
- No pathology at laparoscopy <3 years >2 weeks
- Not on gabapentin/pregabalin
- Not pregnant / planning pregnancy in the next 6 months
- Asked permission to be approached by research staff

Participants approached pre-laparoscopy
- Seen by RT pre-op and given PIS
- Option to contact RT if wish to take part

Visit 1
- Seen at routine hospital follow up visit post-op
- Informed consent
- Screening/eligibility
- Drug randomisation +/- drug dispensing
- Questionnaire

Participants approached post-laparoscopy
- Seen by RT during routine hospital visit and given PIS or sent invitation letter and PIS
- Given option of returning to see RT

Visit 1 (potential additional visit)
- Informed consent
- Screening/eligibility
- Drug randomisation +/- drug dispensing
- Questionnaire

Weekly phone call until dose escalation complete
- Review of AEs
- Option to withdraw

Visit 2 (6 weeks) (additional visit)

Participant
- Collect medication if needed

Research Team
- Review Treatment Diary
- Review AEs
- Option to withdraw

Visit 3 (3 months)

Participant
- Collect medication if needed
- Questionnaire
- ?fMRI

Research Team
- Review Treatment Diary
- Review AEs
- Option to withdraw

Visit 4 (6 months)

Participant
- Questionnaire
- Unblinding

Research Team
- Collect Treatment Diary
- Collect medication
- Review treatment

Contacted (Visits 3 and 4)
- Option to take part in focus group or telephone interview (if consented)

RT = Research Team

JAN12-13
